# Daily Physical Activity Patterns and Their Associations with Cardiometabolic Biomarkers: The Maastricht Study

**DOI:** 10.1249/MSS.0000000000003108

**Published:** 2022-12-27

**Authors:** TUIJA LESKINEN, VALÉRIA LIMA PASSOS, PIETER C. DAGNELIE, HANS H. C. M. SAVELBERG, BASTIAAN E. DE GALAN, SIMONE J. P. M. EUSSEN, COEN D. A. STEHOUWER, SARI STENHOLM, ANNEMARIE KOSTER

**Affiliations:** 1 Department of Public Health, University of Turku and Turku University Hospital, FINLAND; 2 Centre for Population Health Research, University of Turku and Turku University Hospital, FINLAND; 3 Department of Methodology and Statistics, and Care and Public Health Research Institute (CAPHRI), Maastricht University, NETHERLANDS; 4 School of Pharmacy and Biomolecular Sciences, Royal College of Surgeons in Ireland (RCSI), IRELAND; 5 Cardiovascular Research Institute Maastricht School for Cardiovascular Diseases, Maastricht University, NETHERLANDS; 6 Department of Internal Medicine, Maastricht University Medical Center+, NETHERLANDS; 7 Department of Nutrition and Movement Sciences, Maastricht University, Maastricht, NETHERLANDS; 8 NUTRIM School of Nutrition and Translational Research in Metabolism, Maastricht University, Maastricht, NETHERLANDS; 9 Department of Internal Medicine, Radboud University Medical Center, Nijmegen, NETHERLANDS; 10 Department of Epidemiology, Maastricht University, NETHERLANDS; 11 CAPRHI Care and Public Health Research Institute, Maastricht University, NETHERLANDS; 12 Department of social medicine, Maastricht University, NETHERLANDS

**Keywords:** PHYSICAL ACTIVITY, TRAJECTORY MODELING, CARDIOMETABOLIC HEALTH, BIOMARKERS, TYPE 2 DIABETES

## Abstract

**Purpose:**

This study aimed to identify physical activity patterns and examine their association with cardiometabolic biomarkers in a cross-sectional design.

**Methods:**

Overall 6072 participants (mean age, 60.2 yr; SD 8.6 yr, 50% women) from The Maastricht Study provided daily physical activity data collected with thigh-worn activPAL3 accelerometers. The patterns of daily physical activity over weekdays and weekend days were identified by using Group Based Trajectory Modeling. Cardiometabolic biomarkers included body mass index, waist circumference, office blood pressure, glucose, HbA1c, and cholesterol levels. Associations between the physical activity patterns and cardiometabolic outcomes were examined using the analyses of covariance adjusted for sex, age, education, smoking, and diet. Because of statistically significant interaction, the analyses were stratified by type 2 diabetes status.

**Results:**

Overall, seven physical activity patterns were identified: consistently inactive (21% of participants), consistently low active (41%), active on weekdays (15%), early birds (2%), consistently moderately active (7%), weekend warriors (8%), and consistently highly active (6%). The consistently inactive and low active patterns had higher body mass index, waist, and glucose levels compared with the consistently moderately and highly active patterns, and these associations were more pronounced for participants with type 2 diabetes. The more irregular patterns accumulated moderate daily total activity levels but had rather similar cardiometabolic profiles compared with the consistently active groups.

**Conclusions:**

The cardiometabolic profile was most favorable in the consistently highly active group. All patterns accumulating moderate to high levels of daily total physical activity had similar health profile suggesting that the amount of daily physical activity rather than the pattern is more important for cardiometabolic health.

Scientific evidence suggests that adults should do at least 150 to 300 min of moderate-intensity aerobic physical activity; or at least 75 to 150 min of vigorous-intensity aerobic physical activity; or an equivalent combination of moderate- and vigorous-intensity activity throughout the week for substantial health benefits (1). The majority of observational research has so far focused on the average levels of daily or weekly physical activity and their health associations (2). However, such population-average approach overlooks possible heterogeneity in the patterns of physical activity behavior (3–5). For example, accumulating daily physical activity during leisure time is known to promote health whereas occupational, work–time physical activity may not have similar beneficial health effects (referred as physical activity paradox) (6,7). There is also evidence suggesting that physical activity accumulated during only 1 or 2 d·wk^−1^, behavior often referred to as weekend warriors, may be sufficient to reduce the risk for all-cause, cardiovascular, and cancer mortality (8).

The use of accelerometers has enabled researchers to examine daily, hour-by-hour patterns of physical activity in detail (3,9). In previous studies, daily physical activity patterns have been shown to differ by employment status so that weekday patterns are different for low versus highly educated individuals (10), and by age groups as afternoon and evening activity drops mid to old age (11,12). Physical activity patterns have also shown to change after retirement transition when work-related activity reduces (13). The previous studies have also shown that the daily physical activity patterns for workdays and days-off are different (10,14,15). Thus, current evidence suggests the presence of a variable picture on the courses of physical activity not only in volume and in how it is spread over the day, but also the nuances in timing, duration and magnitude of physical activity can diverge between weekdays and weekend days, all of which may be better captured by a model-based clustering technique (16).

The heterogeneity of the unfolding patterns of daily physical activity based on cross-sectional data has been previously captured and described by the use of latent trajectory modeling, and linked to health-related outcomes (14,17,18). For example, among Finnish aging workers, a weekday pattern showing moderate levels of physical activity during usual working hours followed by higher activity levels in the evening was associated with a more favorable health-related physical fitness compared with the most inactive pattern (14). In the National Health and Nutrition Examination Survey population from the United States, the more active trajectories modeled over the entire week, including the pattern of the weekend warriors, were found to have lower odds for cardiometabolic risk factors, such as obesity, high blood pressure, glucose, and triglyceride levels, low high-density lipoprotein (HDL), and, metabolic syndrome, compared with the sedentary trajectory group (18). However, the patterns combining weekdays’ and weekend days’ daily physical activity have not yet been studied.

The current study aimed to fill the research gap by examining the heterogeneity of joint daily physical activity patterns over weekdays and weekend days and their associations with cardiometabolic biomarkers in a large sample of The Maastricht Study population (19). Based on the previous literature, we expect substantive variation in physical activity over weekdays and weekend days and aim to afford some clarity on whether and how such variation is differentially linked to cardiometabolic health. We apply the multivariate version of the model-based clustering technique known as group-based trajectory modeling (GBTM) (20). The GBTM capitalize on the underlying heterogeneity of individual longitudinal behaviors by identifying distinct temporal patterns of changes in one or multiple outcomes (16,20). This novel analytical approach allows for joint latent class modeling to uncover the heterogeneity in weekdays and weekends physical activity patterns while unveiling their cross-linkages and estimating their prevalence.

## METHODS

### Study Population

The present study uses cross-sectional baseline data from The Maastricht Study. The Maastricht Study is an observational prospective population-based cohort study. Its rationale and methodology are described in detail elsewhere (19). In brief, the study focuses on the etiology, pathophysiology, complications, and comorbidities of type 2 diabetes and is characterized by an extensive phenotyping approach. Eligible participants were individuals between 40 and 75 yr of age and living in the southern part of the Netherlands (municipalities Maastricht, Margraten-Eijsden, Meersen, and Valkenburg; Maastricht and Heuvelland in the province of Limburg). Participants were recruited through mass media campaigns and from the municipal registries and the regional Diabetes Patient Registry via mailings. Recruitment was stratified according to known type 2 diabetes status, with an oversampling of individuals with type 2 diabetes, for reasons of efficiency (19).

The Maastricht Study had 7689 participants who completed the baseline measurements between November 2010 and January 2018. The clinical examinations, accelerometer measurements, and questionnaires of each participant were performed within a time window of 3 months.

The Maastricht Study was approved by the institutional medical ethical committee (NL31329.068.10) and the Minister of Health, Welfare, and Sports of the Netherlands (permit no. 131088-105234-PG). All participants gave written informed consent.

### Assessment of Physical Activity

Daily physical activity was measured using the triaxial accelerometer activPAL3 (PAL Technologies, Glasgow, UK). The device was attached directly to the skin during the clinical examinations. The small device was positioned on the front of the right thigh with transparent 3M Tegaderm™ tape, after the device had been waterproofed using a nitrile sleeve. Participants were asked to wear the accelerometer for eight consecutive days, without removing it at any time. To avoid inaccurately identifying nonwear time, participants were asked not to replace the device once removed.

#### Data processing

The raw accelerometer data were uploaded using the activPAL software and processed using customized software written in MATLAB R2018b (MathWorks, Natick, MA) (21). The software determines time spent in three postures, specifically sitting or lying, standing, and stepping. Data of the first measurement day were excluded, because participants performed physical function tests at the research center that day. Hour-by-hour data for posture-based behaviors, i.e., the stepping minutes for each hour of the day, were calculated for each measurement day. We were able to separate the daily data for each weekday (Monday to Friday) and weekend day (Saturday and Sunday). A valid day was defined as over 10 h of daily waking physical activity (sitting, lying, standing or stepping) data. The participants who did not provide at least four valid days or had no measurements on weekend days were excluded from the analysis (*n* = 1617). Overall, the analytical sample of 6072 participants provided 6.5 (SD, 0.7; range, 4–7) valid days. This amount of data is considered as a reliable measure of total and moderate-to-vigorous physical activity (22).

We calculated the daily total physical activity by summing up all the daily stepping minutes for all valid days, and separately for all valid weekdays and all valid weekend days. The mean daily moderate-to-vigorous activity (MVPA) was calculated as the daily minutes with step frequency of ≥100 steps per minute (23) for all valid days, and for all valid weekdays and weekend days separately.

### Cardiometabolic Biomarkers

Detailed description of the general data collection and protocols for the laboratory assessments are reported elsewhere (19). The cardiometabolic biomarkers included measured weight and height for the calculation of body mass index (BMI, kg·m^−2^), waist circumference, office systolic and diastolic blood pressure, and laboratory assessed HbA1c, fasting plasma glucose, total-to-HDL ratio, and triglycerides. A standardized 7-point oral glucose tolerance test (OGTT) was conducted for all participants after an overnight fast. Blood samples were taken at baseline, and 15, 30, 45, 60, 90 and 120 min after ingestion of a 75 g glucose drink. Diabetes status of the participants was assessed by medication use and by the OGTT and dichotomized into the following: participants without type 2 diabetes (normal glucose tolerance, impaired fasting glucose or impaired glucose tolerance) and participants with type 2 diabetes (19).

The number of missing values for the cardiometabolic biomarkers was very low ranging from 1 to 6 missing values per biomarker, except for the 2-h fasting plasma glucose, the number of missing values was 368.

### Covariates

Sex, age, level of education (low, medium, high), smoking status (never, former, current), and diet (Dutch healthy diet index sum score, including alcohol (24)) were assessed with a questionnaire as described earlier (19,25) and used as covariates. These covariates were selected as they have been shown to be associated with physical activity (26,27) as well as with the cardiometabolic biomarkers (28). We used self-reported employment status (working/nonworking), mobility limitations (defined as having difficulty walking 500 m or climbing up a flight of stairs, yes/no) and cardiovascular disease (yes/no) to further characterize the population (19). Cardiovascular disease was defined as a self-reported history of myocardial infarction, cerebrovascular infarction or hemorrhage, percutaneous artery angioplasty, or vascular surgery on the coronary, abdominal, peripheral, or carotid arteries.

### Statistical Analysis

Inferential analyses were conducted in two phases: first we identified the joint physical activity patterns using trajectory modeling and then examined their associations with the cardiometabolic biomarkers.

#### Trajectory modeling

For the trajectory modeling, we first averaged the hour-by-hour physical activity data from all valid weekdays and from all valid weekend days, separately. Then we applied the hourly weekday and weekend day physical activity data to cover the most common waking hours, i.e., from 6:00 am in the morning to 12 midnight, thus excluding the usual night time hours. Finally, the hourly activity minutes of two consecutive hours were averaged for the trajectory modeling.

The presence of distinct subtypes of physical activity behavior for weekdays and weekend days was explored using the GBTM (20). The GBTM is an exploratory tool for recognition and visualization of different patterns of temporal change and, as such, an adequate model for analysis of unobserved heterogeneity in developmental paths (16,20). Models were first run with 1 to 10 activity pattern solutions for weekday and weekend days separately (univariate GBTM) to acquire an idea of the latent heterogeneity of both outcomes. Subsequently, the multivariate version of GBTM was fitted (20).

The choice of the best model was based on model fit criteria and the clinical relevance of the identified activity patterns. Model selection was assisted by the following fit statistics: Akaike Information Criterion, Bayesian Information Criterion, likelihood, average posterior probability of assignment (APPA), odds of correct classification, mismatch between estimated and assigned probabilities and standard deviation of group membership probabilities (see Supplemental Digital Content 1, http://links.lww.com/MSS/C774). Theoretical relevance was judged by visual inspection of the extracted physical activity classes, so as to capture underlying heterogeneity while factoring in their sizes to avoid sparseness in further inferences (customarily the 1% criterion is applied). After settling for the final number of the bivariate daily patterns of physical activity (class enumeration), each participant is classified to one of the classes based on his/her maximum posterior probability of assignment (see Supplemental Digital Content 1 for the output of the GBTM model, http://links.lww.com/MSS/C774). Supplemental Digital Content 1 (http://links.lww.com/MSS/C774) includes all the details of the fit statistics and parameter estimates of the model. Because the mean posterior probability for the assignment to each latent class was over 0.8, we used a classify–analyze strategy for further inferential analyses (classes were treated as known, i.e., handled as deterministic categories). The analyses were conducted using *proc traj* in SAS software (v. 9.4 SAS Institute, Cary, NC) and class enumeration was assisted by the Fit-criteria Assessment Plot (F-CAP) (29) in RStudio software (v. 3.6.3; RStudio, PBC, Boston, MA).

#### Statistical modeling

Descriptive characteristics of the analytical sample and the identified pattern groups are presented as mean values and standard deviations for the continuous variables and percentages for the categorical variables. χ^2^ Test and ANOVA were used for unadjusted groups’ comparisons for categorical and continuous variables, respectively. Adjusted associations between the extracted pattern groups and cardiometabolic biomarkers were examined using the ANCOVA (*proc glm*). For these models, we specifically tested the moderating effects of diabetes, sex and employment status by considering the interaction terms diabetes–group, sex–group, and employment–group. This was done because diabetes was the focus of the Maastricht study, leading an oversampling of individuals with type 2 diabetes. Sex is a well-established moderator in associations between risk factors and cardiovascular health outcomes (28) and a correlate of physical activity (26). Moreover, the participants of The Maastricht Study comprise both working (employed) and nonworking individuals (including individuals retired, not able to work, and not working for other reasons), which may affect both the daily physical activity patterns and the risk factors. In case of significant interactions, subgroups analyses, i.e., stratification by diabetes status, sex and employment status, were conducted to demonstrate the differential associations between pattern groups and biomarkers as a function of type 2 diabetes, sex, and employment status. All models were adjusted for sex, age, education, smoking, and diet. The results are presented as estimated means and their 95% confidence intervals (CI) from the adjusted models unless otherwise stated.

## RESULTS

Characteristics of the study population are shown in Table 1. The mean age of the participants was 60.2 yr (standard deviation [SD], 8.6 yr; range, 40–79 yr), 50% of them were women, 38% had a high educational level, and 43% were employed. The mean BMI was 26.9 kg·m^−2^ (SD, 4.5 kg·m^−2^). In terms of health status, 25% of the participants had type 2 diabetes, 17% had cardiovascular disease, and 21% reported having mobility limitations. The study population (*n* = 6072) was about 2 yr older (*P* < 0.0001), and they had slightly lower BMI (26.9 kg·m^−2^ vs 27.2 kg·m^−2^, *P* = 0.03), waist circumference (95.1 cm vs 96.1 cm, *P* = 0.02), and total-to-HDL ratio (3.58 vs 3.77, *P* < 0.0001) compared with the participants not providing valid physical activity data (*n* = 1617) (Table S3, Supplemental Digital Content 2, Comparison of the cardiometabolic biomarkers among participants included vs excluded from the analyses, http://links.lww.com/MSS/C775).

**TABLE 1:** Characteristics for the study population and by the identified joint physical activity patterns, the Maastricht study. The highest values are marked in bold, the lowest in bold/italic.

	All	Consistently Inactive	Consistently Low Active	Active on Weekdays	Early Birds	Consistently Moderately Active	Weekend Warriors	Consistently Highly Active	P*
*N*	6072	1301	2498	881	138	449	453	352	
Characteristics									
Age: mean (SD), yr	60.2 (8.6)	61.3 (9.0)	60.2 (8.7)	61.9 (7.9)	58.3 (8.2)	***56.9 (7.9)***	***55.9 (7.9)***	61.6 (7.1)	<0.0001
The Dutch Healthy Diet sum score, mean (SD)	84.2 (15.1)	***79.9 (14.8)***	84.6 (14.9)	86.1 (14.7)	85.2 (14.6)	85.5 (15.8)	85.7 (14.9)	**87.6 (14.6)**	<0.0001
Women, %	50	40	55	53	41	52	43	54	<0.0001
Level of education, %									<0.0001
Low	35	**40**	35	38	35	27	***16***	39	
Medium	27	26	27	28	35	27	30	28	
High	38	34	38	34	***30***	46	**54**	33	
Current smoking, %	13	**21**	12	9	10	12	9	***5***	<0.0001
Employed, %	43	37	42	27	**66**	56	**76**	30	<0.0001
Diabetes, %	25	**43**	24	21	18	14	***10***	***11***	<0.0001
Cardiovascular disease, %	17	**24**	18	18	12	***9***	***8***	11	<0.0001
Mobility limitations, %	21	**37**	22	13	14	12	***6***	11	<0.0001
Physical activity									
Daily total physical activity: mean (95% CI), min·d^−1^	117.9 (SD 40.2)	***68.2 (67.2–69.2)***	108.5 (107.7–109.2)	148.1 (146.9–149.4)	175.6 (172.5–178.7)	157.6 (155.8–159.3)	138.1 (136.4–139.9)	**193.3 (191.3–195.2)**	<0.0001**
Daily MVPA: mean (95% CI) min·d^−1^	22.2 (SD 17.6)	***10.3 (9.5–10.0)***	18.4 (17.8–19.0)	28.9 (27.9–29.8)	40.2 (37.9–42.6)	31.6 (30.2–32.9)	32.3 (30.9–33.6)	**44.3 (42.8–45.8)**	≤0.007**

*P values for ANOVA and χ^2^ models. **Estimated means and their 95% CI adjusted with age and sex from the generalized linear model model, *P* value for comparisons between each pattern and the consistently highly active pattern.

### Identification of physical activity patterns

**FIGURE 1:**
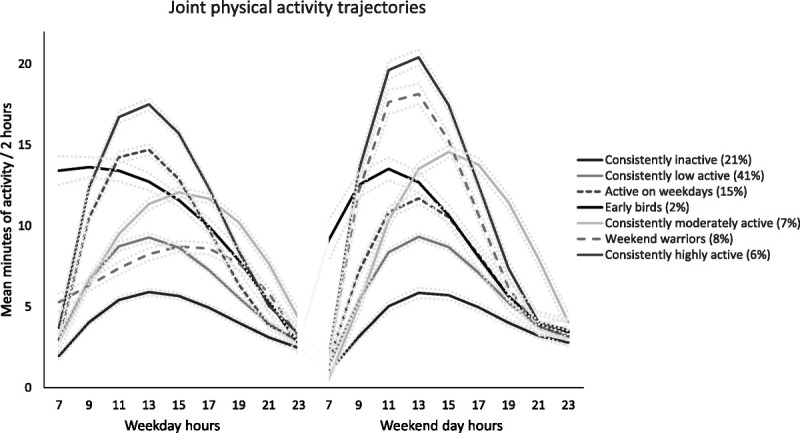
Joint physical activity patterns for weekdays and weekend days with confidence intervals from the multivariate group-based trajectory model.

The fit statistics revealed seven groups to be a good fit for the model (see Supplemental Digital Content 1 for more detailed motivation for this choice, http://links.lww.com/MSS/C774). Figure 1 illustrates the seven joint physical activity patterns over weekdays and weekend days. One fifth of the population (21%) was allocated to *the consistently inactive* pattern group, in which low levels of physical activity were observed throughout the day on both weekdays and weekend days. The largest proportion of the population (41%) was allocated to *the consistently low active* group with a low physical activity on weekdays and weekend days. Overall, 15% of the participants were allocated to *the active on weekdays* group, in which physical activity peaked during midday hours in weekdays but remained at lower level during weekend days. A small group of the participants (2%) were allocated to *the pattern of early birds* in which the physical activity levels were high during the early morning hours, but decreased thereafter on both weekdays and weekend days. Participants in *the consistently moderately activity* group (7%) accumulated moderate levels of physical activity in the afternoon hours in both weekdays and weekend days, whereas *the weekend warriors* (8%) accumulated high physical activity during weekend days only. *The consistently highly active* group (6%) showed the highest activity levels peaking before afternoon hours on both weekdays and weekends.

**FIGURE 2:**
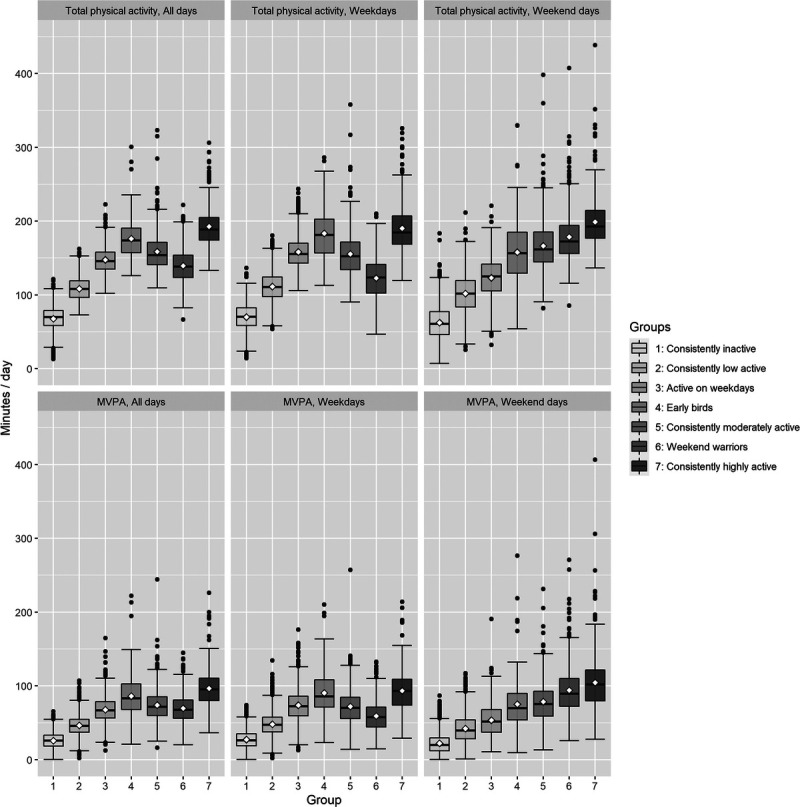
Boxplots of the observed values for daily total and MVPA by the physical activity patterns.

The characteristics for the latent physical activity pattern groups are shown in Table 1. The participants allocated to *the consistently inactive*, *the consistently low activity,* and *the active on weekdays* had a lower education, poorer diet, and poorer health status, when compared with *the consistently highly active* group. On the other hand, the participants allocated to *the weekend warriors* and *the consistently moderately active* groups were younger and had a higher level of education than those in *the consistently highly active* group. *The early birds* and *the weekend warriors* included the highest proportions of employed participants (66% and 76%, respectively). The estimated mean daily total physical activity (i.e., daily time spent stepping) was significantly different for all the pattern groups (*P* < 0.0001 for all comparisons) being the lowest for *the consistently inactive* (68 min·d^−1^; 95% CI, 67–69 min·d^−1^), and *low active* groups (109 min·d^−1^, 95% CI, 108–109 min·d^−1^), and the highest for *the consistently highly active* group (193 min·d^−1^, 95% CI, 191–195 min·d^−1^) (Table 1). Overall, the higher the group’s daily total activity was, the higher was their amount of daily MVPA, except for *the weekend warriors* who accumulated moderate level of total activity and high level of MVPA (Table 1). The observed values for daily total and MVPA for all valid days, weekdays, and weekend days by the pattern groups are shown in Figure 2.

### Physical activity patterns and cardiometabolic biomarkers

Because we found a significant diabetes–group interaction for the majority of the outcomes (BMI, waist circumference, glucose, HbA1c, triglycerides, interaction; *P* < 0.05 for all), the main results are given separately for participants without (Table 2) and with type 2 diabetes (Table 3). Models’ parameter estimates linking pattern groups and other covariates to the cardiometabolic biomarkers are provided in the Supplemental Digital Content 3 (http://links.lww.com/MSS/C776).

**TABLE 2:** Cardiometabolic biomarkers by the physical activity patterns for the participants without type 2 diabetes (*N* = 4545).

	Consistently Inactive		Consistently Low Active		Active on Weekdays		Early Birds		Consistently Moderately Active		Weekend Warriors		Consistently Highly Active (REF)	
*n*	742		1893		696		112		383		406		313	
	Mean	95% CI	Mean	95% CI	Mean	95% CI	Mean	95% CI	Mean	95% CI	Mean	95% CI	Mean	95% CI
BMI, kg·m^−2^	**26.9**	**26.6–27.2**	**26.2**	**26.0–26.4**	**25.3**	**25.0–25.6**	**25.8**	**25.1–26.5**	25.0	24.6–25.4	**25.6**	**25.2–26.0**	24.6	24.2–25.0
Waist, cm	**95.0**	**94.2–95.8**	**92.8**	**92.3–93.3**	**90.7**	**89.9–91.5**	**91.5**	**89.6–93.5**	88.9	87.9–90.0	**90.7**	**89.7–91.8**	88.1	86.9–89.2
HbA1c, %	5.5	5.4–5.5	5.5	5.4–5.5	5.5	5.4–5.5	5.5	5.4–5.5	5.4	5.4–5.5	5.4	5.4–5.5	5.5	5.4–5.5
Fasting glucose, mmol·L^−1^	5.3	5.2–5.3	5.3	5.2–5.3	5.3	5.2–5.3	5.2	5.1–5.3	5.2	5.1–5.3	5.3	5.2–5.3	5.3	5.2–5.3
OGTT 2-hour glucose, mmol·L^−1^	**6.2**	**6.0–6.3**	**6.0**	**5.9–6.0**	5.8	5.7–5.9	5.6	5.2–5.9	5.6	5.4–5.7	5.8	5.6–6.0	5.6	5.4–5.8
Total to HDL ratio	**3.8**	**3.7–3.9**	**3.7**	**3.7–3.8**	3.6	3.5–3.7	3.2	3.0–3.5	3.4	3.3–3.5	3.6	3.4–3.7	3.5	3.3–3.6
Triglycerides, mmol·L^−1^	**1.5**	**1.4–1.5**	**1.4**	**1.3–1.4**	1.3	1.2–1.3	1.2	1.0–1.3	1.2	1.2–1.3	1.3	1.2–1.3	1.2	1.1–1.3
Systolic blood pressure, mm Hg	131.1	129.8–132.2	131.3	130.5–132.1	131.4	130.1–132.7	**135.2**	**132.1–138.4**	130.3	128.6–132.0	131.4	129.7–133.1	130.8	128.9–132.7
Diastolic blood pressure, mm Hg	74.8	74.1–75.5	75.0	74.5–75.5	75.2	74.4–76.0	**76.8**	**75.0–78.7**	74.1	73.1–75.1	75.4	74.4–76.3	74.4	73.3–75.5

Estimated means and their 95% CI from GLM models adjusted for age, sex, education, smoking and diet.

Bolded values indicate values significantly higher than those among the reference group (REF).

**TABLE 3:** Cardiometabolic biomarkers by the physical activity patterns for the participants with type 2 diabetes (*N* = 1487).

	Consistently Inactive		Consistently Low Active		Active on Weekdays		Early Birds		Consistently Moderately Active		Weekend Warriors		Consistently Highly Active (Ref)	
*n*	550		585		181		25		63		46		37	
	Mean	95% CI	Mean	95% CI	Mean	95% CI	Mean	95% CI	Mean	95% CI	Mean	95% CI	Mean	95% CI
BMI, kg·m^−2^	**30.9**	**30.4–31.3**	**29.1**	**28.7–29.6**	**28.3**	**27.6–29.1**	27.9	26.1–29.8	28.3	27.1–29.5	27.1	25.7–28.4	26.8	25.3–28.3
Waist, cm	**107.7**	**106.6–108.9**	**102.7**	**101.6–103.9**	99.8	98.0–101.7	100.1	95.3–105.0	98.9	95.9–102.0	98.8	95.4–102.3	97.1	93.2–101.0
HbA1c, %	**6.9**	**6.8–7.0**	6.7	6.6–6.8	6.6	6.4–6.8	6.8	6.4–7.2	6.8	6.5–7.0	6.5	6.2–6.8	6.5	6.1–6.8
Fasting glucose, mmol·L^−1^	**7.9**	**7.7–8.1**	7.5	7.3–7.7	7.5	7.2–7.8	7.4	6.6–8.2	**7.9**	**7.4–8.4**	7.4	6.9–8.0	7.0	6.3–7.6
OGTT 2-hour glucose, mmol·L^−1^	**14.7**	**14.3–15.1**	**14.4**	**14.0–14.8**	14.2	13.6–14.9	13.2	11.4–14.9	14.4	13.3–15.5	14.3	13.2–15.5	13.0	11.6–14.3
Total to HDL ratio	3.8	3.7–3.9	3.5	3.4–3.6	3.5	3.3–3.7	3.5	3.0–4.0	3.4	3.1–3.6	3.3	3.0–3.6	3.6	3.2–4.0
Triglycerides, mmol·L^−1^	1.9	1.8–2.0	1.6	1.6–1.7	1.6	1.4–1.7	1.3	0.9–1.7	1.7	1.4–1.9	1.5	1.2–1.8	1.6	1.3–2.0
Systolic blood pressure, mm Hg	138.5	136.9–140.3	140.9	139.2–142.5	138.9	136.1–141.7	144.4	137.2–151.6	140.1	135.6–144.7	141.2	136.1–146.3	140.5	134.7–146.2
Diastolic blood pressure, mm Hg	75.8	74.9–76.7	75.6	74.7–76.5	75.6	74.1–77.1	75.6	71.7–79.5	75.7	73.3–78.2	76.3	73.5–79.1	74.6	71.5–77.8

Estimated means and their 95% CI from GLM models adjusted for age, sex, education, smoking and diet.

Bolded values indicate values significantly higher than those among the reference group (REF).

For the participants without type 2 diabetes, all patterns, except *the consistently moderately active,* had 0.7 to 2.3 kg·m^−2^ higher BMI (*P* ≤ 0.006 for all) and 2.6 to 7.0 cm greater waist circumference (*P* ≤ 0.003 for all) compared with *the consistently highly active* group (Table 2). Also, *the active on weekdays*, *the early birds* and *the weekend warriors* had ~2 cm higher waist circumference compared with *the consistently moderately active* group. *The consistently inactive*, *the consistently low active* and *the active on weekdays* groups had ~0.08 mmol·L^−1^ higher plasma fasting glucose values than *the consistently moderately active* group, but they did not differ from that of *the consistently highly active* group. In addition, *the consistently inactive* and *the consistently low active* groups differed from *the consistently highly active* group in terms of higher 2-h glucose, total-to-HDL ratio and triglyceride levels (Table 2). *The early birds* had the highest blood pressure levels (systolic: 135.2 mm Hg, 95% CI 132.1–138.4 and diastolic: 76.8 mm Hg, 95% CI 75.0–78.7) compared with the other patterns.

For the participants with type 2 diabetes, *the consistently inactive* group had significantly higher BMI (30.9 kg·m^−2^; 95% CI, 30.4–31.3 vs 26.9 kg·m^−2^, 95% CI 26.6–27.2), waist (107.7 cm, 95% CI 106.6–108.9 vs 97.1 cm, 95% CI 106.6–108.9), HbA1c (6.9 mmol·L^−1^, 95% CI 6.8–7.0 vs 6.5 mmol·L^−1^, 95% CI 6.1–6.8), fasting glucose (7.9 mmol·L^−1^; 95% CI 7.7–8.1 vs 7.0 mmol·L^−1^, 95% CI, 6.3–7.6 mmol·L^−1^), and 2-h glucose levels (14.7 mmol·L^−1^, 95% CI, 14.3–15.1 vs 13.0 mmol·L^−1^, 95% CI, 11.6–14.3 mmol·L^−1^) compared with *the consistently highly active* group (Table 3). Furthermore, *the consistently inactive* group had higher total-to-HDL ratio and higher triglycerides compared with other pattern groups, except to *the consistently highly active* group. No significant differences between the activity pattern groups and blood pressure levels among the participants with type 2 diabetes were found. Adjustment for employment status did not change the aforementioned results (data not shown).

We observed sex–group interaction with three cardiometabolic outcomes (interaction *P* = 0.05 for BMI, *P* = 0.01 for HbA1c, *P* = 0.002 for 2-h glucose). The results are presented for women in Table S4 and for men in Table S5 (see Supplemental Digital Content 2, Cardiometabolic biomarkers by the physical activity patterns for the female and male participants, http://links.lww.com/MSS/C775). Women in *the consistently inactive* and *the consistently low active* groups had higher BMI, HbA1c and 2-h glucose levels compared with *the consistently highly active* group. For men, each pattern group had higher BMI, and the majority of them had also higher HbA1c and 2-h blood glucose levels compared with *the consistently highly active* group.

We also found a significant employment status–group interaction for BMI (*P* = 0.01), waist circumference (*P* = 0.03), HbA1c (*P* = 0.009), and fasting glucose (*P* = 0.004). The results for nonworking (Table S6) and working (Table S7) participants are given in Supplemental Digital Content 2 (Cardiometabolic biomarkers by the physical activity patterns for the nonworking and working participants, http://links.lww.com/MSS/C775). Among nonworking participants, the three most inactive groups along with *the weekend warriors* had higher BMI, and all groups, except *the consistently moderately active,* had 3.2 to 11.6 cm greater waist circumference (≤0.05 for all) compared with *the consistently highly active* group. *The consistently inactive* and *low active* groups had higher HbA1c and fasting glucose levels compared with *the consistently highly active* group (Table S6, Supplemental Digital Content 2, http://links.lww.com/MSS/C775). For working participants, compared with *the consistently highly active* group, *the consistently inactive* and *low active* groups had higher BMI and fasting glucose, and all groups, except *the consistently moderately active,* had 2.6 to 8.5 cm greater waist circumference (≤0.05 for all) (Table S7, Supplemental Digital Content 2, http://links.lww.com/MSS/C775).

## DISCUSSION

In this study, we aimed to identify joint physical activity patterns over weekdays and weekend days in a large sample of middle-age and older adults as previous evidence suggests that different patterns of physical activity may be found when combining weekday and weekend day data (3,4,15). Overall, seven different activity patterns were extracted: *the consistently inactive* (21% of the participants), *the consistently low active* (41%), *the active on weekdays* (15%), *the early birds* (2%), *the consistently moderately active* (7%), *the weekend warriors* (8%), and *the consistently highly active* (6%). The most favorable cardiometabolic profile was found among *the consistently highly active* pattern group and the poorest cardiometabolic profile was among *the consistently inactive pattern* group. The more irregular patterns showed rather similar cardiometabolic profiles compared with each other and with the consistently active groups.

The identified patterns accumulated daily physical activity differently over weekdays and weekend days and resulted with significantly different daily total physical activity levels. The more consistent patterns accumulated both the lowest (*the consistently inactive* and *low active*) and the highest (*the consistently moderately* and *highly active*) amounts of daily physical activity, whereas the more irregular patterns (*the early birds*, *the active on weekdays* and *weekend warriors*) accumulated moderate-to-high amounts of daily total physical activity. These findings extend the findings from previous studies, which have examined physical activity patterns across the whole week or separately for weekdays and weekend days (5,14,18), that different patterns result with different amounts of total physical activity.

Overall, the majority (62%) of the participants in our study were allocated to *the consistently inactive* and *the consistently low active* pattern groups, which is comparable to the findings from the US population (3). The least active pattern groups showed the highest BMI, waist circumference, and blood glucose levels, when compared especially with *the consistently moderately active* and *the consistently highly active* groups, and this was seen in all subgroups studied. This finding is in line with the well-known dose–response association between physical activity level/volume and health outcomes (2). Our results also complement the previous studies using latent trajectory modeling (14,18), as in these studies, the most active pattern was found to differ from the most sedentary pattern in terms of health-related outcomes. In our study, these differences were more pronounced among the participants with type 2 diabetes (vs no diabetes) and among nonworking people (vs working people). These findings may be explained by the accumulation of the nonworking participants and the participants with type 2 diabetes to the least active groups, and by their higher overall levels of the cardiometabolic biomarkers and other risk factors, such as high age and chronic conditions.

In addition to the consistently active and inactive patterns, we found three more irregular patterns (*the early birds*, *the active on weekdays,* and *the weekend warriors*). These irregular patterns accumulated moderate-to-high levels of daily total activity peaking at different time of the day or week. Overall, these patterns did not differ from each other in terms of cardiometabolic biomarkers. However, they showed higher BMI and waist circumference compared with the consistently active patterns, especially among participants without type 2 diabetes (vs those with type 2 diabetes) and among men (vs women). Most likely these differences between the consistent and irregular patterns may be related to the lower total and MVPA levels among the more irregular patterns. High BMI or type 2 diabetes *per se* may hamper the ability to engage to especially vigorous physical activity (30). Thus, among the participants without type 2 diabetes, the pattern may matter more on the accumulation of MVPA. Also, engagement in vigorous physical activity has been shown to be higher among men than women (26,31), which may explain the less pronounced differences between the active patterns among women. Unfortunately, our data of MVPA minutes were not usable for the GBTM, because the amount of hourly MVPA was often zero or too small, and it accumulated unevenly during the waking hours. Therefore, further studies of the different daily MVPA patterns are warranted.

Interestingly, we identified *the active on weekdays* pattern (15% of the sample), in which physical activity peaked during midday hours on weekdays only. Participants assigned to this class had low activity levels during evenings and weekend days, the habitual times for leisure-time physical activity, what may underlie the rather moderate level of daily total physical activity and higher BMI and waist circumference compared with *the consistently moderately* and/or *highly active* groups. Unfortunately, we were not able to separate whether the peak of the activity was during work-time or leisure time to study more carefully the physical activity paradox (6). However, the subgroup analysis showed higher BMI, waist and glucose levels among the *active on weekdays* group compared with the *consistently moderately/highly active* among the working people which may hint that physical activity accumulated during the usual working hours associates with poorer cardiometabolic health than a pattern that peaks later in the afternoon. However, more studies on daily physical activity patterns during working hours/days and their health-outcomes are needed to elucidate the physical activity paradox (7).

The small group of *early birds*, for which the physical activity level was the highest during the early morning hours on both weekdays and weekend days, did not differ in their cardiometabolic profile from the *consistently highly active* group, except that *the early birds* had higher BMI and waist circumference, and also the highest blood pressure levels among the participants without type 2 diabetes. A similar *early bird* pattern was found in a previous Dutch study (4), in which the activity accumulated during the early morning hours was found to consist of light physical activity, possibly reflecting active commuting. Active commuting has been found to benefit health (32), but the available data did not allow us to tease out the commuting activity.

We were also able to identify the pattern of *weekend warriors* with high levels of physical activity on weekend days only; a similar pattern was also found in the NHANES population (3). However, because of their lower weekday activity levels, *the weekend warriors* did not reach as high daily total physical activity as *the early birds*, *the consistently moderately* and *highly active* groups. This may be the reason behind their higher BMI and waist circumference, compared with the more consistent patterns having higher overall physical activity levels, even though their cardiometabolic biomarker profile was rather similar (which may in turn result from rather high level of MVPA). Our findings are to some extent in agreement with previous ones showing pattern of *weekend warriors* to be associated with lower risk for metabolic syndrome, (18) and both low inflammatory markers (33) and vascular stiffness (34). However, our observations on high BMI and waist circumference among *the weekend warriors* underline that more studies of the potential health-promoting role of *the weekend warrior* type of activity are needed (35).

### Strengths and limitations

This study was conducted among a very large population of middle-age and older men and women from the Maastricht study with an extensive data of accelerometer-measured physical activity behavior in both weekdays and weekend days. For the first time, we were able to study joint weekdays and weekends daily physical activity patterns using GBTM (20). We conducted the trajectory modeling for daily total physical activity minutes to cover all physical activities. Limiting the daily physical activity patterns to a time period of 6:00 to 24:00 for all individuals may have not captured all waking hours, but it was not possible to include all hours of the day for the GBTM analyses because of the nonmovement hours during the normal night time.

Furthermore, assuming the extracted latent trajectories as deterministic groups for additional inferential analyses, i.e., discarding the probabilistic nature of class-assignment, is known to introduce bias in the estimates, specifically an underestimation of effect sizes and associated standard errors (36). However, given the high APPA for all classes (APPA >0.80), such bias is expected to have negligible impact on the findings. The rather small pattern groups in the subgroup analysis, especially among the participants with type 2 diabetes, may have affected the statistical power to detect significant links to the cardiometabolic biomarkers. Lastly, due to the cross-sectional design of this study, we precluded from drawing causal claims. Of note, reverse causality cannot be ruled out to explain the observed associations with physical activity patterns and health outcomes.

## CONCLUSIONS

Heterogeneity in the patterns of physical activity over weekdays and weekend days was detected among middle-age and older adults. The consistent patterns that accumulated high levels of physical activity on both weekdays and weekend days were associated with the most favorable cardiometabolic biomarker profile, whereas *the consistently inactive* and *low active* patterns had the poorest cardiometabolic health. We also found more irregular patterns, namely *the active on weekdays*, *the early birds* and *the weekend warriors*, which accumulated moderate-to-high amounts of daily total activity during certain times of the day or week rather than constantly during the entire week. The cardiometabolic profile among all the patterns accumulating moderate to high levels of daily total physical activity was similar, suggesting that overall, the amount of daily physical activity rather than the pattern is more important for cardiometabolic health. However, because the patterns seem to accumulate different amounts of daily physical activity and MVPA, they may contribute differently to daily energy expenditure. Therefore, further prospective studies and interventions are warranted to examine health associations of different physical activity patterns.

## Supplementary Material

SUPPLEMENTARY MATERIAL
