# Supplementary material for: Daily Physical Activity Patterns and Their Associations with Cardiometabolic Biomarkers: The Maastricht Study
Source: Med Sci Sports Exerc. 2022 Dec 27;55(5):837–46. doi: 10.1249/MSS.0000000000003108 (PMC10090323; doi:10.1249/MSS.0000000000003108)
Supplement: SUPPLEMENTARY MATERIAL [file msse-55-837-s002.docx]

**Supplementary material 1.**

**Fit-Criteria Assessment Plots (FCAP) for trajectory modeling**

Akaike's information criterion (AIC), Bayesian information criterion (BIC) and Likelihood (L)

Average Posterior probability (of assignment), mismatch (between assigned and estimated groups probabilities) and SD (of group membership probabilities)

Odds of correct classification (OCC)

Percentage of individuals belonging to the smallest group

**Brief explanation of the FCAP**

The Fit-Criteria Assessment Plot (FCAP) is an automated visual display of several fit criteria recorded when fitting multi-GBTM for different and increasing numbers of classes. For a closer description of this plot, readers are referred to original publication (Klijn et al. 2017). In a gist, The FCAP combines eight goodness-of-fit and model-adequacy criteria in compact graphs for several user stipulated varying k’s (i.e., number of classes). The basic idea behind the FCAP is that the user can see how indices change by increasing the number of latent trajectories. The visual display of their behavior in such condensed form allows for different criteria to be assessed side by side to reach a well-informed decision. In doing so, it fosters transparency in the process both for the user and the critical appraiser.

With the physical activity data, the FCAP shows some inklings of a plateauing behavior of the information criteria indices (BIC, AIC and likelihood, the top plot), though it reaches a best fit solution at k=9. A plateauing behavior has been shown to be diagnostic for variance under-specification, and that following the information criteria best fit solution is prone to class over extraction (van der Nest et al. 2022). Because GBTM does not allow for classes to have different variance-covariance structure, we paid careful attention to avoid over-extraction. The additional two plots provided with k=8 and k=9 show that although some interesting classes could have been extracted (like the very late on the day physical activity patterns), their sizes were too small to warrant further considerations.

The other fit criteria (the additional plots) were also relatively inconclusive. Note that the minimum average posterior probability (APPA-) remains above the 80% for all explored solutions, and the other ones behave similarly. The 1% rule of thumb for the class size comes in here as a decider (only k=7 was above this threshold).

**References:**

Klijn, S. L., Weijenberg, M. P., Lemmens, P., van den Brandt, P. A., & Lima Passos, V. (2017). Introducing the fit-criteria assessment plot–A visualisation tool to assist class enumeration in group-based trajectory modelling. Statistical methods in medical research, 26(5), 2424-2436.

van der Nest, G., Lima Passos, V., Candel, M. J., & van Breukelen, G. J. (2022). Model fit criteria curve behaviour in class enumeration–a diagnostic tool for model (mis) specification in longitudinal mixture modelling. Journal of Statistical Computation and Simulation, 92(8), 1640-1672.

**The GRoLTS list**

**Item 1: Is the metric of time used in the statistical model reported?**

YES – Physical activity in minutes/2 hours, the details are described in the methods section.

**Item 2: Is the information about the mean and variance of time within a wave?**

NA – Non-applicable (Cross-sectional data with 24 hours measurements, averaged across weekdays and weekends).

**Item 3a: Is the missing data mechanism reported?**

NA (no missing values)

**Item 3b: Is description provided of what variables are related to missing values?**

NA

**Item3c: Is the description provided of how missing values were dealt with?**

NA

**Item 4: Is information about the distribution of observed values included?**

Spaghetti plots of a random sample is presented and the information of the link function for running GBTM is provided (censored normal) – see syntax.

**Item 5: Is the software mentioned?**

YES – *proc traj* in SAS (version 9.4)

**Item 6a: Are alternative specifications of within class variance considered?**

NO – GTBM handles only class invariance (no within-class subject-variability except random noise). The discussion on the need of subject-specific random effects is an ongoing debate, which is beyond the scope of the present paper. All model fitting and selection strategies were conducted in the awareness of the advantages and disadvantages of the GBTM technique, so that extra careful consideration was taken avoid e.g., overextraction, while unveiling sufficient heterogeneity in the data. It should be added that the multivariate approach is expected to obliviate the need of random effects, because of the joint interpretation of classes.

Consider, for instance, the classes ‘*weekend warrior’* and ‘*consistently highly active’*, in particular the weekend plot (on the right in Figure 1). Their average courses were similar in shape and differed marginally in level (what in the univariate model could have been captured by random effects, instead on an extra class). However, their weekdays patterns were substantially different, warranting the selection of this additional class in the joint picture.

**Item 6b:** **are alternative specifications of between-classes variance-covariance structure considered?**

NA – in the GBTM (univariate or multivariate), error variance is assumed to be the same for all classes

**Item 7:** **are alternative shapes and functional forms of the trajectories described?**

YES – the model strategy applied started from the highest possible polynomial order for all classes, which still guaranteed model convergence (in our case cubic). Then the polynomials were pruned down until the best fit model was selected. Final regression parameters are provided.

**Item 8: If covariates have been used, can the analysis be replicated?**

NA – the unconditional model was fitted (without covariates).

**Item 9: Is information about the number of random starts and iteration included?**

YES – the number of iterations is 971.

**Item 10: Are model comparisons and selection tools described (from the statistical perspective)?**

YES – This is the purpose of the FCAP.

**Item 11: Are the total number of fitted models reported, inclusive one class solution?**

YES – This is the purpose of the FCAP.

**Item 12: Are the number of cases and proportion per class reported for each model?**

The FCAP records the smallest proportion for different class solutions.

**Item 13: Is entropy reported?**

No entropy, but the average posterior probability of assignment - APPA (entropy is a function of the posterior probability of assignment) – see FCAP.

**Item 14a:** **Is the plot included with the average mean trajectory of the final solution?**

YES – see Figure 1

**Item 14b: Are additional plots included with estimates means trajectories?**

YES – the trajectories plots for the solutions with k=8 and k=9 joint trajectories are provided to allow for a comparison with the final solution (7 latent trajectories). Of note, the fit criteria, e.g., BIC, suggests that k=9 solution may be the best fit to the data.

- Physical activity daily courses (weekdays and weekends) of the GBTM with solution **k=8** (eight latent classes). The overall figure is similar to the selected one (k=7), with a difference of class 8. This would indicate the presence of a subgroup of individuals who are physically active at the end of the day (both on weekdays and weekend). However, this class was too small (0.1%) to allow for further inferential analyses.


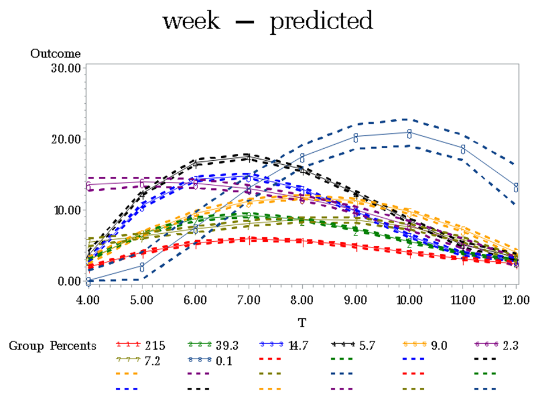

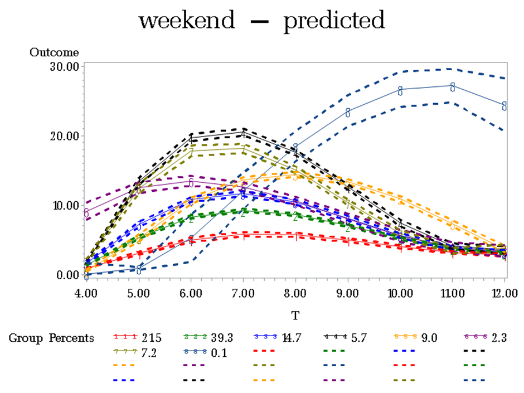


- Physical activity daily courses (weekdays and weekends) of the GBTM with solution **k=9** (nine latent classes). The ninths would be characterised by moderate to high activity levels (marginally lower than the high-activity in the k=7 solution with a right shift on the time line). Yet, here too, the estimated size of the latent class was 0% (convergence issues also emerged, as standard errors could not be computed). All things considered, we settled for k=7.


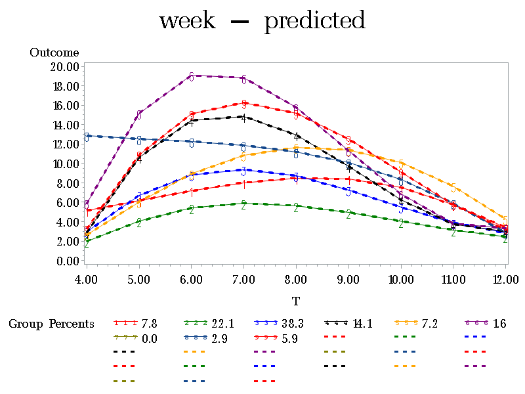

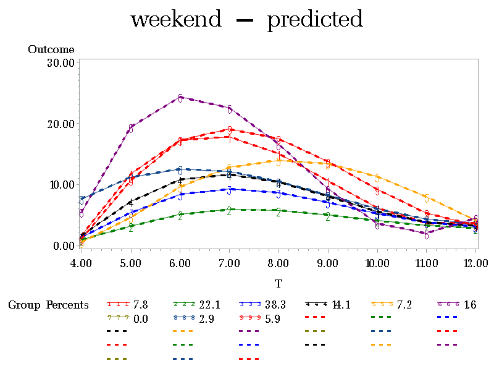


**Item 14c: Are subject-specific trajectories per class provided?**

YES – Spaghetti plots for a random sample of n=300 are available.

**
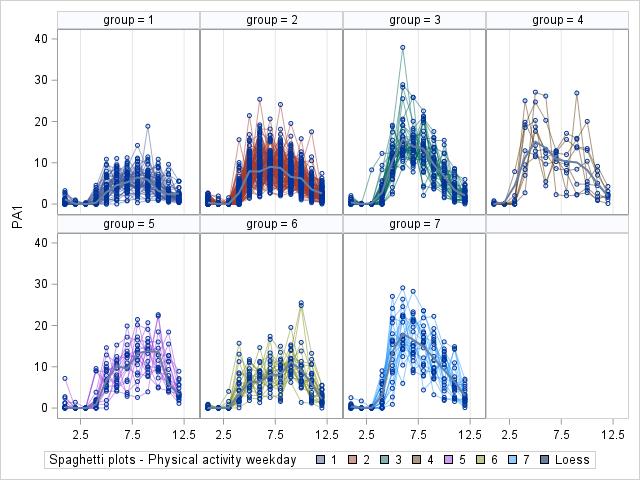
**

**
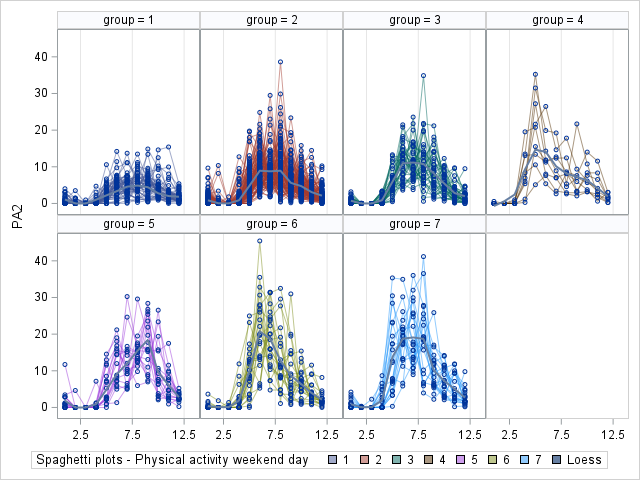
**

Spaghetti plots of a random sample (N=300) of the data with subject-specific physical activity lines for each extracted bivariate latent group.

PA1=weekday physical activity minutes, PA2=weekend day physical activity minutes

**Item 15: Are characteristics of the final class solution numerically described?**

YES - Additional to the physical activity courses (trajectories) plots, we are providing a table with estimated and observed physical activity averages per latent group/time point (See Tables S1 and S2).

**Item 16: Are the syntax files available?**

Yes, the syntax with regression parameters is available as a supplement.

**SAS syntax for the model**

**PROC** **TRAJ** DATA=valid OUTPLOT=OP OUTSTAT=OS OUT=OF OUTEST=OE OUTPLOT2=op2 OUTSTAT2=os2 ITDETAIL;

ID RandomID; VAR bi_stm4-bi_stm12; INDEP T4-T12;

MODEL CNORM; MIN **0**; MAX **60**; NGROUPS **7**; ORDER **3** **3** **3** **3** **3** **3** **3**;

VAR2 bi_we4-bi_we12; INDEP2 t4-t12;

MODEL2 CNORM; MIN2 **0**; MAX2 **60**; NGROUPS **7**; ORDER2 **3** **3** **3** **3** **3** **3** **3**;

MULTGROUPS **7**;

%***TRAJPLOTNEW*** (OP, OS, 'week - predicted');

%***TRAJPLOTNEW*** (OP2, OS2, 'weekend - predicted');

**quit**;

**Maximun Likehood Estimates of the trajectory groups (SAS output)**

Maximum Likelihood Estimates

Model: Censored Normal (CNORM)

Standard T for H0:

Group Parameter Estimate Error Parameter=0 Prob > |T|

1 Intercept -46.52732 1.75451 -26.519 0.0000

Linear 17.80242 0.72513 24.551 0.0000

Quadratic -1.94167 0.09314 -20.846 0.0000

Cubic 0.06607 0.00379 17.419 0.0000

2 Intercept -66.75602 1.38387 -48.239 0.0000

Linear 26.74208 0.57753 46.304 0.0000

Quadratic -2.99420 0.07429 -40.304 0.0000

Cubic 0.10368 0.00302 34.335 0.0000

3 Intercept -85.08494 2.31154 -36.809 0.0000

Linear 34.87178 0.95720 36.431 0.0000

Quadratic -3.99713 0.12259 -32.605 0.0000

Cubic 0.14149 0.00498 28.431 0.0000

4 Intercept -41.47799 5.63845 -7.356 0.0000

Linear 22.03804 2.36674 9.312 0.0000

Quadratic -2.76036 0.31091 -8.878 0.0000

Cubic 0.10251 0.01288 7.958 0.0000

5 Intercept -71.10022 3.78542 -18.783 0.0000

Linear 24.61249 1.57612 15.616 0.0000

Quadratic -2.14725 0.20434 -10.508 0.0000

Cubic 0.05115 0.00833 6.138 0.0000

6 Intercept -149.03969 4.37156 -34.093 0.0000

Linear 62.74061 1.93755 32.381 0.0000

Quadratic -7.48061 0.25635 -29.181 0.0000

Cubic 0.27567 0.01052 26.211 0.0000

7 Intercept -159.51731 3.77423 -42.265 0.0000

Linear 66.78873 1.62395 41.127 0.0000

Quadratic -7.87173 0.21319 -36.924 0.0000

Cubic 0.28608 0.00876 32.662 0.0000

Sigma 4.59619 0.01571 292.521 0.0000

Group membership

1 (%) 21.93608 1.08241 20.266 0.0000

2 (%) 39.51715 0.98988 39.921 0.0000

3 (%) 15.27682 0.87781 17.403 0.0000

4 (%) 2.36312 0.27717 8.526 0.0000

5 (%) 7.62123 0.58322 13.067 0.0000

6 (%) 7.51312 0.55959 13.426 0.0000

7 (%) 5.77249 0.37737 15.297 0.0000

BIC= -304378.1 (N= 109296)

BIC= -304285.6 (N= 6072)

AIC= -304070.8

ll= -304006

**Table S1.** Estimated and observed physical activity averages per latent group/time point for weekday hours.

| **Hour of the day** | **Average 1** | **Average 2** | **Average 3** | **Average 4** | **Average 5** | **Average 6** | **Average 7** | **Model Estimate 1** | **Model Estimate 2** | **Model Estimate 3** | **Model Estimate 4** | **Model Estimate 5** | **Model Estimate 6** | **Model Estimate 7** | **Lower Bound 95% C.I.** | **Upper Bound 95% C.I.** | **Lower Bound 95% C.I.** | **Upper Bound 95% C.I.** | **Lower Bound 95% C.I.** | **Upper Bound 95% C.I.** | **Lower Bound 95% C.I.** | **Upper Bound 95% C.I.** | **Lower Bound 95% C.I.** | **Upper Bound 95% C.I.** | **Lower Bound 95% C.I.** | **Upper Bound 95% C.I.** | **Lower Bound 95% C.I.** | **Upper Bound 95% C.I.** |
| --- | --- | --- | --- | --- | --- | --- | --- | --- | --- | --- | --- | --- | --- | --- | --- | --- | --- | --- | --- | --- | --- | --- | --- | --- | --- | --- | --- | --- |
|  |  |  |  |  |  |  |  |  |  |  |  |  |  |  | **for Mean 1** | **for Mean 1** | **for Mean 2** | **for Mean 2** | **for Mean 3** | **for Mean 3** | **for Mean 4** | **for Mean 4** | **for Mean 5** | **for Mean 5** | **for Mean 6** | **for Mean 6** | **for Mean 7** | **for Mean 7** |
| 7 | 1.39 | 2.42 | 2.39 | 12.66 | 2.47 | 4.55 | 3.13 | 1.96 | 3.01 | 2.95 | 13.42 | 2.81 | 5.28 | 3.70 | 1.77 | 2.15 | 2.81 | 3.21 | 2.61 | 3.29 | 12.53 | 14.31 | 2.29 | 3.33 | 4.73 | 5.83 | 3.19 | 4.22 |
| 9 | 4.22 | 7.27 | 10.70 | 15.39 | 7.14 | 7.76 | 13.02 | 4.05 | 6.65 | 10.49 | 13.63 | 6.52 | 6.32 | 12.37 | 3.84 | 4.25 | 6.42 | 6.89 | 10.21 | 10.77 | 13.00 | 14.26 | 6.17 | 6.88 | 5.98 | 6.67 | 12.05 | 12.68 |
| 11 | 5.41 | 8.84 | 15.10 | 13.04 | 9.31 | 6.92 | 17.04 | 5.42 | 8.74 | 14.24 | 13.40 | 9.50 | 7.38 | 16.73 | 5.21 | 5.64 | 8.46 | 9.02 | 13.90 | 14.58 | 12.75 | 14.06 | 9.13 | 9.86 | 6.98 | 7.78 | 16.34 | 17.11 |
| 13 | 5.56 | 8.76 | 13.65 | 11.69 | 11.14 | 8.14 | 16.71 | 5.91 | 9.28 | 14.70 | 12.73 | 11.35 | 8.25 | 17.51 | 5.71 | 6.11 | 9.01 | 9.55 | 14.38 | 15.03 | 12.20 | 13.26 | 11.01 | 11.70 | 7.87 | 8.63 | 17.15 | 17.86 |
| 15 | 5.36 | 8.39 | 12.87 | 10.91 | 11.77 | 7.50 | 15.68 | 5.67 | 8.66 | 12.88 | 11.59 | 12.07 | 8.72 | 15.71 | 5.48 | 5.86 | 8.45 | 8.88 | 12.61 | 13.15 | 11.21 | 11.97 | 11.77 | 12.38 | 8.38 | 9.06 | 15.43 | 15.99 |
| 17 | 5.04 | 7.48 | 9.77 | 11.40 | 12.07 | 9.21 | 12.27 | 4.94 | 7.27 | 9.76 | 9.97 | 11.68 | 8.60 | 12.34 | 4.74 | 5.15 | 7.11 | 7.44 | 9.53 | 9.99 | 9.47 | 10.47 | 11.38 | 11.99 | 8.24 | 8.95 | 12.08 | 12.61 |
| 19 | 3.95 | 5.75 | 6.58 | 7.92 | 10.39 | 8.49 | 8.84 | 4.01 | 5.55 | 6.37 | 7.88 | 10.21 | 7.69 | 8.43 | 3.79 | 4.22 | 5.39 | 5.71 | 6.11 | 6.63 | 7.18 | 8.58 | 9.87 | 10.54 | 7.30 | 8.08 | 8.11 | 8.75 |
| 21 | 2.56 | 3.61 | 3.71 | 4.76 | 7.41 | 5.30 | 4.97 | 3.11 | 3.94 | 3.85 | 5.34 | 7.68 | 5.83 | 5.07 | 2.93 | 3.28 | 3.80 | 4.09 | 3.61 | 4.09 | 4.61 | 6.08 | 7.33 | 8.02 | 5.47 | 6.20 | 4.71 | 5.43 |
| 23 | 1.81 | 2.28 | 2.38 | 2.27 | 4.12 | 2.67 | 2.83 | 2.45 | 2.83 | 2.98 | 2.68 | 4.27 | 3.11 | 3.32 | 2.26 | 2.64 | 2.68 | 2.99 | 2.71 | 3.25 | 2.04 | 3.32 | 3.81 | 4.74 | 2.71 | 3.50 | 2.87 | 3.76 |

**Table S2.** Estimated and observed physical activity averages per latent group/time point for weekend day hours.

| **Hour of the day** | **Average 1** | **Average 2** | **Average 3** | **Average 4** | **Average 5** | **Average 6** | **Average 7** | **Model Estimate 1** | **Model Estimate 2** | **Model Estimate 3** | **Model Estimate 4** | **Model Estimate 5** | **Model Estimate 6** | **Model Estimate 7** | **Lower Bound 95% C.I.** | **Upper Bound 95% C.I.** | **Lower Bound 95% C.I.** | **Upper Bound 95% C.I.** | **Lower Bound 95% C.I.** | **Upper Bound 95% C.I.** | **Lower Bound 95% C.I.** | **Upper Bound 95% C.I.** | **Lower Bound 95% C.I.** | **Upper Bound 95% C.I.** | **Lower Bound 95% C.I.** | **Upper Bound 95% C.I.** | **Lower Bound 95% C.I.** | **Upper Bound 95% C.I.** |
| --- | --- | --- | --- | --- | --- | --- | --- | --- | --- | --- | --- | --- | --- | --- | --- | --- | --- | --- | --- | --- | --- | --- | --- | --- | --- | --- | --- | --- |
|  |  |  |  |  |  |  |  |  |  |  |  |  |  |  | **for Mean 1** | **for Mean 1** | **for Mean 2** | **for Mean 2** | **for Mean 3** | **for Mean 3** | **for Mean 4** | **for Mean 4** | **for Mean 5** | **for Mean 5** | **for Mean 6** | **for Mean 6** | **for Mean 7** | **for Mean 7** |
| 7 | 0.46 | 0.87 | 1.00 | 7.76 | 0.45 | 1.41 | 1.58 | 0.95 | 1.35 | 1.60 | 9.11 | 0.54 | 1.77 | 1.83 | 0.95 | 0.96 | 1.30 | 1.40 | 1.45 | 1.74 | 7.85 | 10.38 | 0.38 | 0.70 | 1.50 | 2.04 | 1.56 | 2.11 |
| 9 | 2.77 | 5.13 | 7.04 | 15.36 | 4.49 | 11.03 | 11.94 | 3.14 | 5.37 | 7.16 | 12.52 | 5.05 | 12.11 | 13.40 | 2.92 | 3.36 | 5.14 | 5.61 | 6.79 | 7.52 | 11.76 | 13.28 | 4.50 | 5.59 | 11.40 | 12.83 | 12.89 | 13.90 |
| 11 | 4.85 | 8.49 | 11.26 | 13.51 | 9.80 | 19.18 | 21.07 | 5.03 | 8.37 | 10.83 | 13.52 | 10.34 | 17.65 | 19.63 | 4.71 | 5.35 | 8.12 | 8.61 | 10.47 | 11.18 | 12.82 | 14.23 | 9.85 | 10.84 | 16.90 | 18.39 | 19.08 | 20.17 |
| 13 | 5.38 | 8.98 | 11.14 | 11.02 | 12.41 | 17.73 | 19.94 | 5.86 | 9.32 | 11.70 | 12.70 | 13.52 | 18.15 | 20.41 | 5.54 | 6.17 | 9.09 | 9.55 | 11.35 | 12.05 | 12.07 | 13.33 | 13.10 | 13.94 | 17.52 | 18.78 | 19.93 | 20.90 |
| 15 | 5.41 | 8.83 | 10.70 | 9.93 | 16.61 | 15.26 | 18.22 | 5.72 | 8.69 | 10.53 | 10.66 | 14.57 | 15.27 | 17.47 | 5.44 | 6.00 | 8.48 | 8.90 | 10.20 | 10.87 | 10.05 | 11.27 | 14.19 | 14.94 | 14.82 | 15.72 | 17.09 | 17.86 |
| 17 | 4.54 | 6.93 | 8.08 | 8.23 | 14.98 | 9.93 | 11.38 | 4.97 | 7.11 | 8.21 | 8.08 | 13.78 | 10.68 | 12.53 | 4.71 | 5.23 | 6.90 | 7.32 | 7.86 | 8.56 | 7.31 | 8.85 | 13.37 | 14.18 | 10.27 | 11.08 | 12.15 | 12.90 |
| 19 | 3.58 | 4.95 | 5.44 | 6.15 | 9.91 | 6.08 | 7.36 | 4.01 | 5.26 | 5.68 | 5.65 | 11.46 | 6.18 | 7.39 | 3.79 | 4.24 | 5.06 | 5.46 | 5.33 | 6.03 | 4.76 | 6.54 | 10.98 | 11.94 | 5.65 | 6.72 | 6.90 | 7.87 |
| 21 | 2.32 | 3.11 | 3.32 | 4.00 | 6.98 | 3.64 | 4.02 | 3.21 | 3.80 | 3.84 | 4.00 | 7.98 | 3.61 | 4.05 | 3.04 | 3.38 | 3.65 | 3.94 | 3.59 | 4.10 | 3.30 | 4.69 | 7.47 | 8.50 | 3.23 | 4.00 | 3.65 | 4.45 |
| 23 | 1.75 | 2.24 | 2.33 | 2.26 | 4.25 | 2.69 | 2.60 | 2.79 | 3.11 | 3.20 | 3.44 | 4.04 | 3.71 | 3.53 | 2.57 | 3.01 | 2.93 | 3.29 | 2.90 | 3.51 | 2.61 | 4.26 | 3.47 | 4.61 | 3.20 | 4.21 | 3.01 | 4.06 |
