# Supplementary material for: Daily Physical Activity Patterns and Their Associations with Cardiometabolic Biomarkers: The Maastricht Study
Source: Med Sci Sports Exerc. 2022 Dec 27;55(5):837–46. doi: 10.1249/MSS.0000000000003108 (PMC10090323; doi:10.1249/MSS.0000000000003108)
Supplement: SUPPLEMENTARY MATERIAL [file msse-55-837-s003.docx]

**Supplementary material 2.**

**Table S3.** Comparison of the cardiometabolic biomarkers among participants included vs. excluded from the analyses, The Maastricht Study population.

| **Biomarker** | **Study population with physical activity data, N=6072** | | **Excluded population with no valid physical activity data, N=1617** | | **P-value** |
| --- | --- | --- | --- | --- | --- |
|  | **Mean** | **95% CI** | **Mean** | **95% CI** |  |
| **Age,** years | 60.2 | 60.0-60.4 | 58.5 | 58.0-58.9 | <0.0001 |
| **Body mass index**, kg/m2 | 26.9 | 26.8-27.1 | 27.2 | 27.0-27.5 | 0.03 |
| **Waist circumference,** cm | 95.1 | 94.8-95.5 | 96.1 | 95.4-96.7 | 0.02 |
| **HBA1c,** % | 5.78 | 5.76-5.81 | 5.83 | 5.79-5.88 | 0.07 |
| **Fasting plasma glucose,** mmol/L | 5.93 | 5.89-5.97 | 5.94 | 5.86-6.02 | 0.84 |
| **OGTT 2-hour glucose**, mmol/L | 7.67 | 7.56-7.77 | 7.46 | 7.26-7.66 | 0.07 |
| **Total to HDL ratio** | 3.58 | 3.56-3.61 | 3.77 | 3.70-3.83 | <0.0001 |
| **Triglycerides,** mmol/L | 1.42 | 1.39-1.43 | 1.41 | 1.37-1.45 | 0.66 |
| **Systolic blood pressure,** mmHg | 133.9 | 133.4-134.3 | 133.4 | 132.5-134.3 | 0.36 |
| **Diastolic blood pressure**, mmHg | 75.4 | 75.2-75.7 | 75.9 | 75.4-76.4 | 0.12 |

Means and 95% CI and p-values derived from independent t-test

**Table S4.** Cardiometabolic biomarkers by the physical activity patterns for the female participants (N=3034).

|  | **Consistently inactive** | | **Consistently low active** | | **Active on weekdays** | | **Early birds** | | **Consistently moderately active** | | **Weekend warriors** | | **Consistently highly active**  **(REF)** | |
| --- | --- | --- | --- | --- | --- | --- | --- | --- | --- | --- | --- | --- | --- | --- |
| n | 517 | | 1383 | | 464 | | 56 | | 235 | | 197 | | 191 | |
|  | **Mean** | 95% CI | **Mean** | 95% CI | **Mean** | 95% CI | **Mean** | 95% CI | **Mean** | 95% CI | **Mean** | 95% | **Mean** | 95% CI |
| **Body mass index, kg/m2** | **28.1** | **27.7-28.5** | **26.2** | **25.9-26.5** | 25.0 | 24.5-25.4 | 25.5 | 24.4-26.7 | 24.7 | 24.1-25.3 | **25.2** | **24.5-25.8** | 24.3 | 23.6-24.9 |
| **HBA1c, %** | **5.8** | **5.8-5.9** | **5.6** | **5.6-5.7** | 5.6 | 5.5-5.7 | 5.5 | 5.4-5.7 | 5.5 | 5.4-5.6 | 5.5 | 5.4-5.6 | 5.5 | 5.4-5.6 |
| **OGTT 2-hour glucose, mmol/L** | **7.9** | **7.6-8.3** | **7.0** | **6.8-7.2** | 6.6 | 6.2-6.9 | 6.4 | 5.5-7.3 | 6.2 | 5.7-6.6 | 6.6 | 6.2-7.1 | 6.3 | 5.8-6.8 |

Estimated means and their 95% CI from GLM models adjusted for age, education, smoking and diet.

Bolded values indicate values significantly higher than those among the reference group (REF).

**Table S5.** Cardiometabolic biomarkers by the physical activity patterns for the male participants (N=3029).

|  | **Consistently inactive** | | **Consistently low active** | | **Active on weekdays** | | **Early birds** | | **Consistently moderately active** | | **Weekend warriors** | | **Consistently highly active**  **(REF)** | |
| --- | --- | --- | --- | --- | --- | --- | --- | --- | --- | --- | --- | --- | --- | --- |
| n | 787 | | 1115 | | 417 | | 82 | | 214 | | 256 | | 161 | |
|  | **Mean** | 95% CI | **Mean** | 95% CI | **Mean** | 95% CI | **Mean** | 95% CI | **Mean** | 95% CI | **Mean** | 95% CI | **Mean** | 95% CI |
| **Body mass index,** kg/m2 | **28.6** | **28.3-28.9** | **27.4** | **27.2-27.7** | **26.8** | **26.4-27.2** | **26.5** | **25.6-27.4** | **26.3** | **25.7-26.8** | **26.4** | **25.9-26.9** | 25.4 | 24.8-26.1 |
| **HBA1c,** % | **6.2** | **6.2-6.3** | **5.9** | **5.9-6.0** | **5.8** | **5.7-5.9** | **5.9** | **5.7-6.1** | **5.9** | **5.8-6.0** | 5.7 | 5.6-5.8 | 5.7 | 5.5-5.8 |
| **OGTT 2-hour glucose,** mmol/L | **9.7** | **9.3-10.0** | **8.4** | **8.1-8.7** | **7.9** | **7.4-8.3** | 7.1 | 6.1-8.1 | **7.7** | **7.1-8.3** | **7.5** | **6.9-8.0** | 6.2 | 5.5-6.9 |

Estimated means and their 95% CI from GLM models adjusted for age, education, smoking and diet.

Bolded values indicate values significantly higher than those among the reference group (REF).

**Table S6.** Cardiometabolic biomarkers by the physical activity patterns for the non-working participants (N=3234).

|  | **Consistently inactive** | | **Consistently low active** | | **Active on weekdays** | | **Early birds** | | **Consistently moderately active** | | **Weekend warriors** | | **Consistently highly active**  **(REF)** | |
| --- | --- | --- | --- | --- | --- | --- | --- | --- | --- | --- | --- | --- | --- | --- |
| n | 745 | | 1318 | | 599 | | 43 | | 192 | | 103 | | 234 | |
|  | **Mean** | 95% CI | **Mean** | 95% CI | **Mean** | 95% CI | **Mean** | 95% CI | **Mean** | 95% CI | **Mean** | 95% CI | **Mean** | 95% CI |
| **Body mass index,** kg/m^2^ | **28.6** | **28.3-28.9** | **26.7** | **26.4-26.9** | **25.7** | **25.3-26.0** | 25.8 | 24.5-27.0 | 25.0 | 24.4-25.6 | **26.2** | **25.3-27.0** | 24.5 | 24.0-25.1 |
| **Waist,** cm | **100.8** | **99.9-101.7** | **95.0** | **94.3-95.8** | **92.3** | **90.0-93.3** | **93.4** | **90.0-96.9** | 90.1 | 88.4-91.7 | **93.4** | **91.1-95.6** | 89.1 | 87.6-90.7 |
| **HBA1c,** % | **6.1** | **6.1-6.2** | **5.8** | **5.8-5.9** | **5.7** | **5.7-5.8** | 5.8 | 5.5-6.0 | 5.7 | 5.6-5.9 | 5.7 | 5.5-5.8 | 5.6 | 5.5-5.7 |
| **Fasting glucose,** mmol/L | **6.5** | **6.3-6.6** | **5.9** | **5.8-6.0** | 5.7 | 5.6-5.9 | 5.7 | 5.3-6.2 | 5.7 | 5.5-5.9 | 5.8 | 5.5-6.1 | 5.6 | 5.3-5.8 |

Estimated means and their 95% CI from GLM models adjusted for age, sex, education, smoking and diet.

Bolded values indicate values significantly higher than those among the reference group (REF).

**Table S7.** Cardiometabolic biomarkers by the physical activity patterns for the working participants (N=2418).

|  | **Consistently inactive** | | **Consistently low active** | | **Active on weekdays** | | **Early birds** | | **Consistently moderately active** | | **Weekend warriors** | | **Consistently highly active**  **(REF)** | |
| --- | --- | --- | --- | --- | --- | --- | --- | --- | --- | --- | --- | --- | --- | --- |
| n | 438 | | 1005 | | 222 | | 85 | | 242 | | 326 | | 100 | |
|  | **Mean** | 95% CI | **Mean** | 95% CI | **Mean** | 95% CI | **Mean** | 95% CI | **Mean** | 95% CI | **Mean** | 95% CI | **Mean** | 95% CI |
| **Body mass index,** kg/m^2^ | **27.7** | **27.3-28.1** | **26.8** | **26.6-27.1** | 26.0 | 25.4-26.5 | 26.3 | 25.5-27.2 | 25.6 | 25.1-26.2 | 25.8 | 25.3-26.3 | 25.2 | 24.4-26.0 |
| **Waist,** cm | **97.0** | **95.9-98.1** | **94.3** | **93.6-95.1** | **91.9** | **90.3-93.4** | **92.6** | **90.2-95.1** | 90.0 | 88.5-91.4 | **91.1** | **89.8-92.4** | 88.5 | 86.3-90.8 |
| **HBA1c,** % | **5.8** | **5.7-5.9** | 5.7 | 5.6-5.8 | 5.7 | 5.6-5.8 | 5.7 | 5.5-5.8 | 5.6 | 5.5-5.8 | 5.5 | 5.4-5.6 | 5.6 | 5.4-5.7 |
| **Fasting glucose,** mmol/L | **5.9** | **5.8-6.1** | **5.7** | **5.6-5.8** | **5.7** | **5.5-5.9** | 5.5 | 5.2-5.8 | 5.6 | 5.4-5.8 | 5.4 | 5.2-5.6 | 5.4 | 5.1-5.6 |

Estimated means and their 95% CI from GLM models adjusted for age, sex, education, smoking and diet.

Bolded values indicate values significantly higher than those among the reference group (REF).
