# Supplementary material for: Daily Physical Activity Patterns and Their Associations with Cardiometabolic Biomarkers: The Maastricht Study
Source: Med Sci Sports Exerc. 2022 Dec 27;55(5):837–46. doi: 10.1249/MSS.0000000000003108 (PMC10090323; doi:10.1249/MSS.0000000000003108)
Supplement: SUPPLEMENTARY MATERIAL [file msse-55-837-s004.docx]

**Supplementary material 3. Models’ parameter estimates linking the patterns to all cardiometabolic biomarkers for the participants without type 2 diabetes and participants with type 2 diabetes.**

**Table S8.** Models’ parameter estimates for BMI.

|  | **Participants without type 2 diabetes** | | | | **Participants with type 2 diabetes** | | | |
| --- | --- | --- | --- | --- | --- | --- | --- | --- |
| **Parameter** | **Estimate** | **Standard** | **t Value** | **Pr > \|t\|** | **Estimate** | **Standard** | **t Value** | **Pr > \|t\|** |
|  |  | **Error** |  |  |  | **Error** |  |  |
| **Intercept** | 25.088555 | 0.5724128 | 43.83 | <.0001 | 35.83644 | 1.4563492 | 24.61 | <.0001 |
| **Consistently inactive** | 2.2739585 | 0.258205 | 8.81 | <.0001 | 4.1051609 | 0.7843549 | 5.23 | <.0001 |
| **Consistently low active** | 1.5895374 | 0.2323586 | 6.84 | <.0001 | 2.3464626 | 0.7782376 | 3.02 | 0.003 |
| **Active on weekdays** | 0.7276454 | 0.2577711 | 2.82 | 0.005 | 1.544811 | 0.8289662 | 1.86 | 0.063 |
| **Early birds** | 1.1623277 | 0.4240739 | 2.74 | 0.006 | 1.1357059 | 1.2058125 | 0.94 | 0.346 |
| **Consistently moderately active** | 0.3944289 | 0.2907646 | 1.36 | 0.175 | 1.5263896 | 0.9514193 | 1.6 | 0.109 |
| **Weekend warriors** | 0.9775173 | 0.2888007 | 3.38 | 7E-04 | 0.2735918 | 1.0046706 | 0.27 | 0.785 |
| **Consistently highly active** | 0 | . | . | . | 0 | . | . | . |
| **Men** | 0.7176386 | 0.1226988 | 5.85 | <.0001 | -1.0955127 | 0.2856993 | -3.83 | 1E-04 |
| **Women** | 0 | . | . | . | 0 | . | . | . |
| **Age** | 0.006318 | 0.0071814 | 0.88 | 0.379 | -0.0862419 | 0.0167652 | -5.14 | <.0001 |
| **Diet** | -0.0282185 | 0.0040675 | -6.94 | <.0001 | -0.0624188 | 0.0088229 | -7.07 | <.0001 |
| **Low education** | 1.5004009 | 0.1402731 | 10.7 | <.0001 | 1.3333105 | 0.3043367 | 4.38 | <.0001 |
| **Medium education** | 0.6347579 | 0.1393228 | 4.56 | <.0001 | 0.7720414 | 0.3357566 | 2.3 | 0.022 |
| **High education** | 0 | . | . | . | 0 | . | . | . |
| **Non-smoker** | 0.4505063 | 0.1922161 | 2.34 | 0.019 | 1.7091258 | 0.4077176 | 4.19 | <.0001 |
| **Former smokers** | 1.0181939 | 0.1892129 | 5.38 | <.0001 | 2.1585277 | 0.3792048 | 5.69 | <.0001 |
| **Smoker** | 0 | . | . | . | 0 | . | . | . |

**Table S9.** Models’ parameter estimates for waist circumference.

|  | **Participants without type 2 diabetes** | | | | **Participants with type 2 diabetes** | | | |
| --- | --- | --- | --- | --- | --- | --- | --- | --- |
|  |  |  |  |  |  |  |  |  |
| **Parameter** | **Estimate** | **Standard** | **t Value** | **Pr > \|t\|** | **Estimate** | **Standard** | **t Value** | **Pr > \|t\|** |
|  |  | **Error** |  |  |  | **Error** |  |  |
| **Intercept** | 81.82732 | 1.5687134 | 52.16 | <.0001 | 113.29 | 3.7934145 | 29.86 | <.0001 |
| **Consistently inactive** | 6.964694 | 0.7074281 | 9.85 | <.0001 | 10.64318 | 2.0430426 | 5.21 | <.0001 |
| **Consistently low active** | 4.743636 | 0.6367864 | 7.45 | <.0001 | 5.653581 | 2.0271084 | 2.79 | 0.0054 |
| **Active on weekdays** | 2.631174 | 0.7064034 | 3.72 | 0.0002 | 2.761594 | 2.1592435 | 1.28 | 0.2011 |
| **Early birds** | 3.494078 | 1.1621397 | 3.01 | 0.0027 | 3.032593 | 3.1408311 | 0.97 | 0.3344 |
| **Consistently moderately active** | 0.873901 | 0.7968171 | 1.1 | 0.2728 | 1.819012 | 2.4782021 | 0.73 | 0.4631 |
| **Weekend warriors** | 2.678667 | 0.7914286 | 3.38 | 0.0007 | 1.741275 | 2.6169081 | 0.67 | 0.5059 |
| **Consistently highly active** | 0 | . | . | . | 0 | . | . | . |
| **Men** | 9.561591 | 0.3361283 | 28.45 | <.0001 | 4.581082 | 0.7441731 | 6.16 | <.0001 |
| **Women** | 0 | . | . | . | 0 | . | . | . |
| **Age** | 0.132201 | 0.0196638 | 6.72 | <.0001 | -0.119086 | 0.0436689 | -2.73 | 0.0065 |
| **Diet** | -0.103559 | 0.011143 | -9.29 | <.0001 | -0.183453 | 0.0229814 | -7.98 | <.0001 |
| **Low education** | 3.133679 | 0.38444 | 8.15 | <.0001 | 2.93699 | 0.7927188 | 3.7 | 0.0002 |
| **Medium education** | 1.357632 | 0.3817405 | 3.56 | 0.0004 | 1.67228 | 0.8745594 | 1.91 | 0.0561 |
| **High education** | 0 | . | . | . | 0 | . | . | . |
| **Non-smoker** | 0.462294 | 0.5267574 | 0.88 | 0.3802 | 2.15551 | 1.0619993 | 2.03 | 0.0426 |
| **Former smokers** | 2.354197 | 0.5184652 | 4.54 | <.0001 | 4.771237 | 0.9877309 | 4.83 | <.0001 |
| **Smoker** | 0 | . | . | . | 0 | . | . | . |

**Table S10.** Models’ parameter estimates for HbA1c.

|  | **Participants without type 2 diabetes** | | | | **Participants with type 2 diabetes** | | | |
| --- | --- | --- | --- | --- | --- | --- | --- | --- |
| **Parameter** | **Estimate** | **Standard** | **t Value** | **Pr > \|t\|** | **Estimate** | **Standard** | **t Value** | **Pr > \|t\|** |
|  |  | **Error** |  |  |  | **Error** |  |  |
| **Intercept** | 4.8311627 | 0.056327 | 85.77 | <.0001 | 7.383019 | 0.329646 | 22.4 | <.0001 |
| **Consistently inactive** | 0.0017761 | 0.0253952 | 0.07 | 0.9442 | 0.4738712 | 0.1773563 | 2.67 | 0.0076 |
| **Consistently low active** | 0.0068132 | 0.0228613 | 0.3 | 0.7657 | 0.2451625 | 0.1759854 | 1.39 | 0.1638 |
| **Active on weekdays** | 0.022545 | 0.0253582 | 0.89 | 0.374 | 0.1601644 | 0.1874373 | 0.85 | 0.393 |
| **Early birds** | 0.0231963 | 0.0417181 | 0.56 | 0.5782 | 0.3319262 | 0.2726452 | 1.22 | 0.2237 |
| **Consistently moderately active** | -0.002237 | 0.028604 | -0.08 | 0.9377 | 0.3311644 | 0.2151244 | 1.54 | 0.1239 |
| **Weekend warriors** | -0.020449 | 0.0284272 | -0.72 | 0.472 | 0.0232071 | 0.2271647 | 0.1 | 0.9186 |
| **Consistently highly active** | 0 | . | . | . | 0 | . | . | . |
| **Men** | -0.000555 | 0.0120693 | -0.05 | 0.9633 | 0.1097241 | 0.064646 | 1.7 | 0.0899 |
| **Women** | 0 | . | . | . | 0 | . | . | . |
| **Age** | 0.012102 | 0.0007062 | 17.14 | <.0001 | -0.012399 | 0.0038028 | -3.26 | 0.0011 |
| **Diet** | -8.94E-05 | 0.0004005 | -0.22 | 0.8234 | -0.003518 | 0.0019955 | -1.76 | 0.0781 |
| **Low education** | 0.0282153 | 0.013804 | 2.04 | 0.041 | 0.2044405 | 0.068887 | 2.97 | 0.0031 |
| **Medium education** | -0.016263 | 0.0137063 | -1.19 | 0.2355 | 0.1143057 | 0.0759174 | 1.51 | 0.1324 |
| **High education** | 0 | . | . | . | 0 | . | . | . |
| **Non-smoker** | -0.15224 | 0.0189109 | -8.05 | <.0001 | -0.04785 | 0.0922847 | -0.52 | 0.6042 |
| **Former smokers** | -0.12993 | 0.0186133 | -6.98 | <.0001 | -0.022446 | 0.0857767 | -0.26 | 0.7936 |
| **Smoker** | 0 | . | . | . | 0 | . | . | . |

**Table S11.** Models’ parameter estimates for fasting glucose.

|  | **Participants without type 2 diabetes** | | | | **Participants with type 2 diabetes** | | | |
| --- | --- | --- | --- | --- | --- | --- | --- | --- |
| **Parameter** | **Estimate** | **Standard** | **t Value** | **Pr > \|t\|** | **Estimate** | **Standard** | **t Value** | **Pr > \|t\|** |
|  |  | **Error** |  |  |  | **Error** |  |  |
| **Intercept** | 4.604863218 | 0.0790795 | 58.23 | <.0001 | 9.145843404 | 0.6240176 | 14.66 | <.0001 |
| **Consistently inactive** | 0.022591703 | 0.0356635 | 0.63 | 0.5265 | 0.894202398 | 0.33609 | 2.66 | 0.0079 |
| **Consistently low active** | 0.02562598 | 0.0321011 | 0.8 | 0.4247 | 0.562846247 | 0.3334621 | 1.69 | 0.0917 |
| **Active on weekdays** | 0.016077946 | 0.0356119 | 0.45 | 0.6517 | 0.549372185 | 0.3554499 | 1.55 | 0.1224 |
| **Early birds** | -0.02848621 | 0.0585869 | -0.49 | 0.6268 | 0.481784231 | 0.5166743 | 0.93 | 0.3513 |
| **Consistently moderately active** | -0.05379481 | 0.0401699 | -1.34 | 0.1806 | 0.972580315 | 0.4076655 | 2.39 | 0.0172 |
| **Weekend warriors** | -0.00164877 | 0.0398983 | -0.04 | 0.967 | 0.469630564 | 0.4304816 | 1.09 | 0.2755 |
| **Consistently highly active** | 0 | . | . | . | 0 | . | . | . |
| **Men** | 0.224878316 | 0.0169442 | 13.27 | <.0001 | 0.395469576 | 0.1224894 | 3.23 | 0.0013 |
| **Women** | 0 | . | . | . | 0 | . | . | . |
| **Age** | 0.013839272 | 0.0009913 | 13.96 | <.0001 | -0.03075238 | 0.0071835 | -4.28 | <.0001 |
| **Diet** | -0.00348243 | 0.0005617 | -6.2 | <.0001 | -0.01019008 | 0.0037814 | -2.69 | 0.0071 |
| **Low education** | 0.024042034 | 0.0193776 | 1.24 | 0.2148 | 0.199463177 | 0.1305416 | 1.53 | 0.1268 |
| **Medium education** | 0.00467084 | 0.0192426 | 0.24 | 0.8082 | 0.063411908 | 0.143974 | 0.44 | 0.6597 |
| **High education** | 0 | . | . | . | 0 | . | . | . |
| **Non-smoker** | -0.02929625 | 0.0265552 | -1.1 | 0.27 | 0.447453929 | 0.1747135 | 2.56 | 0.0105 |
| **Former smokers** | 0.054350091 | 0.026137 | 2.08 | 0.0376 | 0.469422083 | 0.1624859 | 2.89 | 0.0039 |
| **Smoker** | 0 | . | . | . | 0 | . | . | . |

**Table S12.** Models’ parameter estimates for OGTT 2-hour glucose.

|  | **Participants without type 2 diabetes** | | | | **Participants with type 2 diabetes** | | | |
| --- | --- | --- | --- | --- | --- | --- | --- | --- |
| **Parameter** | **Estimate** | **Standard** | **t Value** | **Pr > \|t\|** | **Estimate** | **Standard** | **t Value** | **Pr > \|t\|** |
|  |  | **Error** |  |  |  | **Error** |  |  |
| **Intercept** | 3.1582225 | 0.25440613 | 12.41 | <.0001 | 11.247666 | 1.34138474 | 8.39 | <.0001 |
| **Consistently inactive** | 0.5848034 | 0.11476003 | 5.1 | <.0001 | 1.7437351 | 0.69797925 | 2.5 | 0.0126 |
| **Consistently low active** | 0.3711788 | 0.10327616 | 3.59 | 0.0003 | 1.4538718 | 0.68888832 | 2.11 | 0.0351 |
| **Active on weekdays** | 0.2212985 | 0.11456611 | 1.93 | 0.0535 | 1.2741597 | 0.73529286 | 1.73 | 0.0834 |
| **Early birds** | -0.0240803 | 0.18847843 | -0.13 | 0.8983 | 0.1884565 | 1.11649052 | 0.17 | 0.866 |
| **Consistently moderately active** | -0.0119405 | 0.12922977 | -0.09 | 0.9264 | 1.4596086 | 0.86060462 | 1.7 | 0.0902 |
| **Weekend warriors** | 0.2027704 | 0.12835595 | 1.58 | 0.1142 | 1.3573732 | 0.87622896 | 1.55 | 0.1217 |
| **Consistently highly active** | 0 | . | . | . | 0 | . | . | . |
| **Men** | -0.1329495 | 0.05452251 | -2.44 | 0.0148 | -0.3394338 | 0.26307054 | -1.29 | 0.1972 |
| **Women** | 0 | . | . | . | 0 | . | . | . |
| **Age** | 0.0454092 | 0.00318958 | 14.24 | <.0001 | 0.0395612 | 0.01567479 | 2.52 | 0.0118 |
| **Diet** | -0.0063283 | 0.00180731 | -3.5 | 0.0005 | -0.0125193 | 0.00825112 | -1.52 | 0.1295 |
| **Low education** | 0.2996476 | 0.06233991 | 4.81 | <.0001 | 0.495644 | 0.28006426 | 1.77 | 0.0771 |
| **Medium education** | 0.1829424 | 0.06193372 | 2.95 | 0.0032 | 0.3384043 | 0.30653727 | 1.1 | 0.2699 |
| **High education** | 0 | . | . | . | 0 | . | . | . |
| **Non-smoker** | 0.1947824 | 0.08543731 | 2.28 | 0.0227 | 0.4056737 | 0.38108859 | 1.06 | 0.2873 |
| **Former smokers** | 0.3560808 | 0.08409338 | 4.23 | <.0001 | -0.0685499 | 0.35539492 | -0.19 | 0.8471 |
| **Smoker** | 0 | . | . | . | 0 | . | . | . |

**Table S13.** Models’ parameter estimates for total-to-HDL ratio.

|  | **Participants without type 2 diabetes** | | | | **Participants with type 2 diabetes** | | | |
| --- | --- | --- | --- | --- | --- | --- | --- | --- |
| **Parameter** | **Estimate** | **Standard** | **t Value** | **Pr > \|t\|** | **Estimate** | **Standard** | **t Value** | **Pr > \|t\|** |
|  |  | **Error** |  |  |  | **Error** |  |  |
| **Intercept** | 4.12218959 | 0.1679821 | 24.54 | <.0001 | 5.55245123 | 0.36189709 | 15.34 | <.0001 |
| **Consistently inactive** | 0.35040034 | 0.07571615 | 4.63 | <.0001 | 0.18246045 | 0.19490914 | 0.94 | 0.3494 |
| **Consistently low active** | 0.28503094 | 0.06815209 | 4.18 | <.0001 | -0.0502844 | 0.19338899 | -0.26 | 0.7949 |
| **Active on weekdays** | 0.12797915 | 0.07562057 | 1.69 | 0.0906 | -0.1150778 | 0.20599487 | -0.56 | 0.5765 |
| **Early birds** | -0.2172229 | 0.12438169 | -1.75 | 0.0808 | -0.0804021 | 0.29963971 | -0.27 | 0.7885 |
| **Consistently moderately active** | -0.0561982 | 0.08528269 | -0.66 | 0.51 | -0.2272917 | 0.23642397 | -0.96 | 0.3365 |
| **Weekend warriors** | 0.08681344 | 0.08470581 | 1.02 | 0.3055 | -0.2691823 | 0.24965672 | -1.08 | 0.2811 |
| **Consistently highly active** | 0 | . | . | . | 0 | . | . | . |
| **Men** | 0.61623808 | 0.03597932 | 17.13 | <.0001 | 0.36773497 | 0.07099516 | 5.18 | <.0001 |
| **Women** | 0 | . | . | . | 0 | . | . | . |
| **Age** | -0.011397 | 0.0021046 | -5.42 | <.0001 | -0.0294768 | 0.00416608 | -7.08 | <.0001 |
| **Diet** | -0.0017236 | 0.00119264 | -1.45 | 0.1485 | -0.0028012 | 0.00219246 | -1.28 | 0.2016 |
| **Low education** | 0.1295467 | 0.0411475 | 3.15 | 0.0017 | 0.13617498 | 0.07562649 | 1.8 | 0.072 |
| **Medium education** | 0.04308356 | 0.04086087 | 1.05 | 0.2918 | 0.03104112 | 0.0834342 | 0.37 | 0.7099 |
| **High education** | 0 | . | . | . | 0 | . | . | . |
| **Non-smoker** | -0.32533 | 0.05642378 | -5.77 | <.0001 | -0.248897 | 0.10131623 | -2.46 | 0.0141 |
| **Former smokers** | -0.2780219 | 0.05553418 | -5.01 | <.0001 | -0.0915643 | 0.09423092 | -0.97 | 0.3314 |
| **Smoker** | 0 | . | . | . | 0 | . | . | . |

**Table S14.** Models’ parameter estimates for triglycerides.

|  | **Participants without type 2 diabetes** | | | | **Participants with type 2 diabetes** | | | |
| --- | --- | --- | --- | --- | --- | --- | --- | --- |
| **Parameter** | **Estimate** | **Standard** | **t Value** | **Pr > \|t\|** | **Estimate** | **Standard** | **t Value** | **Pr > \|t\|** |
|  |  | **Error** |  |  |  | **Error** |  |  |
| **Intercept** | 1.4056759 | 0.1179356 | 11.92 | <.0001 | 3.4418812 | 0.3242452 | 10.62 | <.0001 |
| **Consistently inactive** | 0.2640194 | 0.0531565 | 4.97 | <.0001 | 0.3094033 | 0.1746307 | 1.77 | 0.0767 |
| **Consistently low active** | 0.1804435 | 0.0478483 | 3.77 | 0.0002 | 0.0131314 | 0.1732687 | 0.08 | 0.9396 |
| **Active on weekdays** | 0.0873963 | 0.0530894 | 1.65 | 0.0998 | -0.063122 | 0.1845631 | -0.34 | 0.7324 |
| **Early birds** | -0.02779 | 0.0873222 | -0.32 | 0.7503 | -0.328247 | 0.2684651 | -1.22 | 0.2217 |
| **Consistently moderately active** | 0.046159 | 0.059873 | 0.77 | 0.4408 | 0.0184907 | 0.2118263 | 0.09 | 0.9305 |
| **Weekend warriors** | 0.0634252 | 0.0595069 | 1.07 | 0.2866 | -0.127593 | 0.2236823 | -0.57 | 0.5685 |
| **Consistently highly active** | 0 | . | . | . | 0 | . | . | . |
| **Men** | 0.1663599 | 0.0252682 | 6.58 | <.0001 | 0.0341332 | 0.0636088 | 0.54 | 0.5916 |
| **Women** | 0 | . | . | . | 0 | . | . | . |
| **Age** | 0.0011783 | 0.001478 | 0.8 | 0.4254 | -0.01949 | 0.0037326 | -5.22 | <.0001 |
| **Diet** | -0.0036 | 0.0008375 | -4.3 | <.0001 | -0.007992 | 0.0019644 | -4.07 | <.0001 |
| **Low education** | 0.0889453 | 0.0288897 | 3.08 | 0.0021 | 0.1116153 | 0.0677583 | 1.65 | 0.0997 |
| **Medium education** | 0.0242963 | 0.0286942 | 0.85 | 0.3972 | 0.028475 | 0.0747537 | 0.38 | 0.7033 |
| **High education** | 0 | . | . | . | 0 | . | . | . |
| **Non-smoker** | -0.171264 | 0.0396149 | -4.32 | <.0001 | -0.0632 | 0.0907753 | -0.7 | 0.4864 |
| **Former smokers** | -0.110291 | 0.0389894 | -2.83 | 0.0047 | 0.0869074 | 0.0844271 | 1.03 | 0.3035 |
| **Smoker** | 0 | . | . | . | 0 | . | . | . |

**Table S15.** Models’ parameter estimates for office systolic blood pressure.

|  | **Participants without type 2 diabetes** | | | | **Participants with type 2 diabetes** | | | |
| --- | --- | --- | --- | --- | --- | --- | --- | --- |
| **Parameter** | **Estimate** | **Standard** | **t Value** | **Pr > \|t\|** | **Estimate** | **Standard** | **t Value** | **Pr > \|t\|** |
|  |  | **Error** |  |  |  | **Error** |  |  |
| **Intercept** | 101.34415 | 2.50763895 | 40.41 | <.0001 | 116.94083 | 5.61373984 | 20.83 | <.0001 |
| **Consistently inactive** | 0.2547799 | 1.1310976 | 0.23 | 0.8218 | -1.8903956 | 3.02089527 | -0.63 | 0.5316 |
| **Consistently low active** | 0.4755945 | 1.01792988 | 0.47 | 0.6404 | 0.399618 | 2.99727953 | 0.13 | 0.894 |
| **Active on weekdays** | 0.5892143 | 1.12920596 | 0.52 | 0.6018 | -1.5764817 | 3.19264812 | -0.49 | 0.6215 |
| **Early birds** | 4.4296889 | 1.85771294 | 2.38 | 0.0171 | 3.9031358 | 4.64399367 | 0.84 | 0.4008 |
| **Consistently moderately active** | -0.5312561 | 1.27373697 | -0.42 | 0.6766 | -0.3460458 | 3.67646707 | -0.09 | 0.925 |
| **Weekend warriors** | 0.5756972 | 1.26512312 | 0.46 | 0.6491 | 0.7189571 | 3.8693128 | 0.19 | 0.8526 |
| **Consistently highly active** | 0 | . | . | . | 0 | . | . | . |
| **Men** | 7.2750013 | 0.53737756 | 13.54 | <.0001 | 3.7725959 | 1.10062872 | 3.43 | 0.0006 |
| **Women** | 0 | . | . | . | 0 | . | . | . |
| **Age** | 0.4964452 | 0.03143693 | 15.79 | <.0001 | 0.4330903 | 0.0647561 | 6.69 | <.0001 |
| **Diet** | -0.0760875 | 0.01781129 | -4.27 | <.0001 | -0.102019 | 0.03398139 | -3 | 0.0027 |
| **Low education** | 1.8822144 | 0.61460509 | 3.06 | 0.0022 | -0.1544909 | 1.17283737 | -0.13 | 0.8952 |
| **Medium education** | 1.268409 | 0.61028827 | 2.08 | 0.0377 | -0.7515551 | 1.29310505 | -0.58 | 0.5612 |
| **High education** | 0 | . | . | . | 0 | . | . | . |
| **Non-smoker** | 2.2196855 | 0.84203105 | 2.64 | 0.0084 | 3.8056706 | 1.57115576 | 2.42 | 0.0156 |
| **Former smokers** | 3.3131382 | 0.82885726 | 4 | <.0001 | 4.3708561 | 1.46067967 | 2.99 | 0.0028 |
| **Smoker** | 0 | . | . | . | 0 | . | . | . |

**Table S16.** Models’ parameter estimates for office diastolic blood pressure.

|  | **Participants without type 2 diabetes** | | | | **Participants with type 2 diabetes** | | | |
| --- | --- | --- | --- | --- | --- | --- | --- | --- |
| **Parameter** | **Estimate** | **Standard** | **t Value** | **Pr > \|t\|** | **Estimate** | **Standard** | **t Value** | **Pr > \|t\|** |
|  |  | **Error** |  |  |  | **Error** |  |  |
| **Intercept** | 80.93849169 | 1.46616503 | 55.2 | <.0001 | 98.78655853 | 3.04532445 | 32.44 | <.0001 |
| **Consistently inactive** | 0.43123192 | 0.66120281 | 0.65 | 0.5143 | 1.15539394 | 1.63876604 | 0.71 | 0.4809 |
| **Consistently low active** | 0.63041924 | 0.59505178 | 1.06 | 0.2895 | 0.94016128 | 1.62595504 | 0.58 | 0.5632 |
| **Active on weekdays** | 0.82281675 | 0.66009009 | 1.25 | 0.2126 | 0.97823879 | 1.731938 | 0.56 | 0.5723 |
| **Early birds** | 2.44421646 | 1.08594631 | 2.25 | 0.0245 | 0.91931745 | 2.5192595 | 0.36 | 0.7152 |
| **Consistently moderately active** | -0.25433968 | 0.74458089 | -0.34 | 0.7327 | 1.07655263 | 1.99439863 | 0.54 | 0.5894 |
| **Weekend warriors** | 0.9750056 | 0.73954469 | 1.32 | 0.1874 | 1.61858423 | 2.09901299 | 0.77 | 0.4408 |
| **Consistently highly active** | 0 | . | . | . | 0 | . | . | . |
| **Men** | 4.35884628 | 0.31415893 | 13.87 | <.0001 | 2.62618659 | 0.59706571 | 4.4 | <.0001 |
| **Women** | 0 | . | . | . | 0 | . | . | . |
| **Age** | -0.09584885 | 0.01837698 | -5.22 | <.0001 | -0.37030399 | 0.03512869 | -10.54 | <.0001 |
| **Diet** | -0.05459548 | 0.01041178 | -5.24 | <.0001 | -0.04822101 | 0.01843412 | -2.62 | 0.009 |
| **Low education** | 1.02720197 | 0.3593365 | 2.86 | 0.0043 | 0.08462105 | 0.63623723 | 0.13 | 0.8942 |
| **Medium education** | 0.45457266 | 0.35680868 | 1.27 | 0.2027 | -0.49015002 | 0.70147968 | -0.7 | 0.4848 |
| **High education** | 0 | . | . | . | 0 | . | . | . |
| **Non-smoker** | 1.31426374 | 0.49264691 | 2.67 | 0.0077 | 2.90006962 | 0.85231578 | 3.4 | 0.0007 |
| **Former smokers** | 2.00109474 | 0.48492809 | 4.13 | <.0001 | 3.2263363 | 0.79238505 | 4.07 | <.0001 |
| **Smoker** | 0 | . | . | . | 0 | . | . | . |
